# Integrative gene duplication and genome-wide analysis characterize Peroxin11 gene family in wheat

**DOI:** 10.1186/s12864-026-12771-2

**Published:** 2026-04-11

**Authors:** Heba T. Ebeed

**Affiliations:** 1 Botany and Microbiology Department, Faculty of Science, Damietta University, Damietta, 34517 Egypt; 2 National Biotechnology Network of Expertise (NBNE), Academy of Scientific Research and Technology (ASRT), Cairo, Egypt; 3 Council of Education and Scientific Research Policy (CESRP), Qualitative Councils Sector, ASRT, Cairo, Egypt

**Keywords:** Peroxisome, Wheat, PEX11, Gene family, Genome-wide, Gene duplication, Gene expression

## Abstract

Peroxisomes are essential organelles for plant development and stress adaptation, yet the Peroxin11 (*PEX11*) gene family that mediates peroxisome proliferation remains uncharacterized in wheat (*Triticum aestivum* L.), a key polyploid cereal. This study presents the first comprehensive genome-wide identification and integrative analysis of the wheat *PEX11* genes (*TaPEX11*), identifying 12 loci encoding 13 protein isoforms due to alternative splicing at one locus. Chromosomal mapping revealed uneven distribution, while protein analyses confirmed a conserved Pfam05648 domain with multiple transmembrane helices; some *TaPEX11* members showed divergent features suggestive of specialized roles. Phylogenetic and synteny analyses indicated family expansion through segmental duplications under strong purifying selection. Promoter analysis uncovered diverse *cis*‑elements associated with hormone signalling, development, and abiotic stress responses. Using RNA‑seq data and qRT‑PCR, I demonstrate that distinct *TaPEX11* genes are inducible under drought, with higher expression in the tolerant genotype Masr‑3 compared to the sensitive Sakha‑94. This transcriptional response was associated with increased catalase activity and indicators of oxidative stress response in Masr‑3. Co‑expression network and Gene Ontology analyses linked *TaPEX11* genes to photosynthesis, gluconeogenesis, and peroxisomal homeostasis. Overall, the findings highlight the evolutionary diversification and genotype‑specific regulation of wheat *PEX11* genes under drought and provides candidates genes and regulatory elements for future functional studies and breeding programs targeting drought tolerance.

**Supplementary Information:**

The online version contains supplementary material available at 10.1186/s12864-026-12771-2.

## Introduction

Peroxisomes are metabolic organelles with many functions in plants, laying central roles in lipid degradation, photorespiration, and detoxification of reactive oxygen species (ROS) [1, 2]. Their biogenesis and dynamic regulation require the involvement of peroxin (PEX) proteins, and among them, the PEX11 family is the most relevant in membrane elongation, fission, and proliferation [3]. The *PEX11* gene family is evolutionarily conserved across eukaryotes and typically occur as multiple isoforms with partially specialized functions, as demonstrated in *Arabidopsis thaliana* (e.g., *AtPEX11A-E*) [4].

Functional studies in several plant species demonstrated that *PEX11* members contribute to stress adaptation; for example, *OsPEX11* in rice enhances salt stress tolerance by regulating Na^+^/K^+^ homeostasis and antioxidant defence [5], underscoring the link between peroxisome dynamics and stress adaptation. However, such functional and regulatory insights remain largely limited to few diploid or model species. In contrast, a comprehensive genome-wide and evolutionary characterization of the *PEX11* gene family in wheat (*Triticum aestivum* L.) is still lacking. Wheat is one of the most important crops for global food security, however production is under pressure from climate-induced stresses [6, 7]. As a hexaploid species with A, B, and D sub-genomes, wheat harbors expanded gene families that have undergone complex evolutionary histories involving subfunctionalized or neofunctionalized [8]. Previous work by Cross et al. [3] identified 11 putative *PEX11* homologs in wheat; however, improved genomic resources are now available that enable more refined assessment of gene copy number, structural variation, and evolutionary mechanisms driving the expansions of *PEX11* in plants particularly in polyploids, for example segmental duplication, purifying selection, and syntenic conservation, remain poorly documented.

Drought stress is emphasized in this study because it represents one of the most severe constraints on wheat productivity worldwide and is tightly linked to oxidative stress and peroxisome-mediated ROS homeostasis [6, 7, 9]. Despite the central role of peroxisomes in redox balance, the transcriptional regulation of wheat *PEX11* genes under drought, and their potential genotype-specific responses, have not been systematically explored. Therefore, this study represents the first genome-wide identification and integrative characterization of the *PEX11* gene family in hexaploid wheat. The specific objectives were to (i) perform a genome-wide identification and characterization of the *PEX11* gene family in hexaploid wheat using updated genomic resources; (ii) elucidate their structural features, evolutionary expansion, and syntenic relationships; and (iii) investigate their expression patterns during development and under drought stress to infer potential functional diversification. By integrating evolutionary genomics with stress-responsive transcriptomic and physiological analyses, this work aims to clarify the role of PEX11-mediated peroxisome dynamics in wheat drought resilience.

## Materials and methods

### Identification and characterization of *TaPEX11* genes

#### Identification and nomenclature of *TaPEX11* genes

Identifying PEX11 in wheat was done using *Arabidopsis thaliana* PEX11 (TAIR, http://www.Arabidopsis.org/) through BLASTp (e-value < 1e-5) against wheat database (genome assembly: IWGSC) at EnsemblPlants (https://plants.ensembl.org/Triticum_aestivum/Info/Index) (Supplementary Table S1). All wheat PEX11 proteins retrieved from BLAST searches were accepted only if they contained the conserved domains corresponding to the *Arabidopsis* PEX11 proteins. Multiple sequence alignments were subsequently used to confirm the presence of these conserved regions. Retrieved sequences in wheat were corrected when a portion of protein was missing due to incorrect gene model prediction. Sequences showing large truncations and that could not be completed by further BLAST searches were excluded. A total of 12 *TaPEX11* gene loci were identified and mapped to nine wheat chromosomes belonging to the A, B, and D subgenomes. These loci encode 13 predicted PEX11 protein isoforms, owing to alternative splicing at a single locus. To ensure a consistent and biologically meaningful nomenclature, *TaPEX11* genes were named sequentially (*TaPEX11-1* to *TaPEX11-12*) according to their homoeologous grouping and subgenome distribution, as inferred from chromosomal location and phylogenetic relationships. Within each homoeologous set, genes from the A, B, and D subgenomes were assigned consecutive numbers. Additional loci outside complete triads were numbered subsequently. This approach facilitates comparison among homoeologous copies while maintaining consistency across subgenomes. One locus on chromosome 4 A (TraesCS4A02G442900) generates two transcript isoforms, which encode two protein variants designated TaPEX11-7.1 and TaPEX11-7.2. All other *TaPEX11* loci encode a single protein isoform. Decimal suffixes were used to denote splice variants originating from the same genomic locus, following standard wheat gene-family nomenclature conventions.

#### Characterization of *TaPEX11* genes

The putative *TaPEX11* were next submitted to the NCBI-CDD server (https://www.ncbi.nlm.nih.gov/cdd/) (Supplementary Table S2, Figure S1) and Pfam (v35.0, http://Pfam.xfam.org), (Supplementary Figure S2), and visualization was done by TBtools. ExPASy online tools (http://web.expasy.org/protparam/) predicted the molecular weight (Mw), isoelectric point (pI), and grand average of hydropathicity (GRAVY) of the identified proteins. ClustalW was used to multiple sequence alignments (MSA) of the identified wheat PEX11 proteins. Motifs were predicted and visualized by an online tool MEME (v5.5.1, http://meme-suite.org/). The parameters were given as: the maximum number of motifs was 10, the width from 5 to 50 residues. Motif Scan (https://myhits.sib.swiss/cgi-bin/motif_scan, Table S3) was used for the identification of defined motifs.

### Predictions of TaPEX11 3D structures

Through homology modeling, the Swiss-Model interactive platform (https://swissmodel.expasy.org/) was used to predict the 3D structures of the TaPEX11 proteins. Template structures with sequence similarity at least 85% were selected to define experimentally the tertiary structures of TaPEX11 proteins. The templates were selected based on the highest GMQE being close to 1. The generated 3D models were further validated using the Ramachandran plot. Template identifiers, sequence identity %, alignment coverage, and GMQE score for each model are provided in Supplementary Table S4.

### Phylogenetic analyses of TaPEX11 proteins

Multiple sequence alignments (MSA) of the identified PEX11 proteins of wheat were performed using the ClustalW tool then, geneDoc software [10] was used to visualize the conserved sequences of the TaPEX11 family. MSA of PEX11 protein sequences in different species as *Arabidopsis thaliana* [11] and *Oryza sativa* [12] were performed and the phylogenetic trees were constructed by Maximum likelihood method with MEGA 11 software [13] software, with the bootstrap value set to 1000 and modified by iTol online tool (http://ITOL.embl.de). The gene identifiers of PEX11 in different species are shown in Supplementary Table S5. The Phylogenetic tree for the PEX11 in wheat was also constructed using the above methods.

### Gene structure and chromosomal mapping of *TaPX11* genes

These predicted *TaPEX11* genes' exon–intron organizations were analysed using genomic and CDS sequences from Ensembl Plants (https://plants.ensembl.org/Triticum_aestivum/Info/Index), and the Gene Structure Display Server (GSDS) online tool (v2.0, http://gsds.gao-lab.org/), was used to graphically display them. *TaPEX11*'s gene annotation data was taken from the IWGSC v2.1 GFF3 file. Using the online program for creating the genetic map MG2C_v2.1 (http://mg2c.iask.in/mg2c_v2.1/), the physical map was created using the start and end location information about *TaPEX11* in correspondence chromosomes.

### Gene duplication and collinearity of *TaPEX11*

Synonymous (Ks) and nonsynonymous substitution (Ka) rates were calculated with TBtools [14], as previously described [15]. For each gene pair, the approximate divergence time (T, million years ago, Mya) of the duplication events for each paralogous gene pair was estimated using the mean Ks values from T = Ks/2 λ, in which the mean synonymous substitution rate (λ) for wheat is 6 = 10^–9^ [16, 17].

Gene duplication within the wheat chromosomes were illustrated using Circos plot [18]. To trace the homology of the *PEX11* gene family between wheat and other species, the Dual Systeny Plotter software (https://github.com/CJ-Chen/TBtools) was used for mapping intergenomic collinearity analysis with TBtools [14].

### *Cis*-regulatory element analyses

To gain further understanding of possible *cis*-regulatory elements in the *TaPEX11* gene promoter regions, 2000 bp genomic sequences upstream the transcription start sites of *TaPEX11* genes were retrieved from the Ensembl Plants database (http://plants.ensembl.org/Triticum_aestivum/Info/Index/). Different possible cis-regulatory elements present in the aforementioned sequences were predicted using PlantCARE online tool (http://bioinformatics.psb.ugent.be/webtools/plantcare/html/) [19] and graphically represented using TBtools software (v. 2.152).

### Networking and gene ontology analysis of co-expressed genes and prediction of *TaPEX11* regulation by transcription factors

The co-expressing genes with the *TaPEX11* genes in *T. aestivum* were identified at the threshold filter > 0.9 using the Pearson correlation coefficient (PCC) through WheatOmics 1.0 (http://wheatomics.sdau.edu.cn/coexpression/index.html). Expression data from wheat multi-tissues PRJEB25639 including 14 studies (PRJNA396738, PRJNA407398, PRJNA427246, PRJNA471426, PRJNA477934, PRJNA485741, PRJEB25639, PRJEB5314, PRJEB5135, PRJEB22854, PRJEB7795, PRJNA322418, and PRJNA358808) have been used to conduct the co-expression analysis. Functional annotation and gene ontology (GO) enrichment of co-expressed transcripts were performed using the GOEnrichment tool in Triticeae-Gene Tribe (http://wheat.cau.edu.cn/TGT/, [20]. After predicting the regulatory transcription factors (TF) that target the *PEX11* genes in wheat using the Wheat Regulatory Network (http://bioinfo.sibs.ac.cn/Wheat-RegNet/), a network of TF enrichment was created based on these findings.

### Transcriptome analyses of the *TaPEX11* gene family

The RNA-Seq data were obtained from the Sequence Read Archive database (https://www.ncbi.nlm.nih.gov/), WheatOmics 1.0 (http://wheatomics.sdau.edu.cn/), and Wheat URGI (Unité de Recherche Génomique-Info) (http://www.wheat-expression.com/). Information on accession numbers and on samples is listed in Table S6. The expression profiles of *TaPEX11* genes in five tissues (root, stem, leaf, spike, and grain) at three developmental stages were obtained from WheatOmics, while publicly available RNA-seq data of stress (heat, saline, drought, and cold) were obtained from Wheat URGI and the NCBI. The expression levels of *TaPEX11* genes were then computed and normalized using log2 based TPM (transcripts per million) values. log₂ TPM values were normalized within each dataset and subsequently scaled across comparable samples using quantile normalization to ensure consistency of expression patterns across conditions.

### Plant growth conditions and stress treatment

**Fig. 1:**
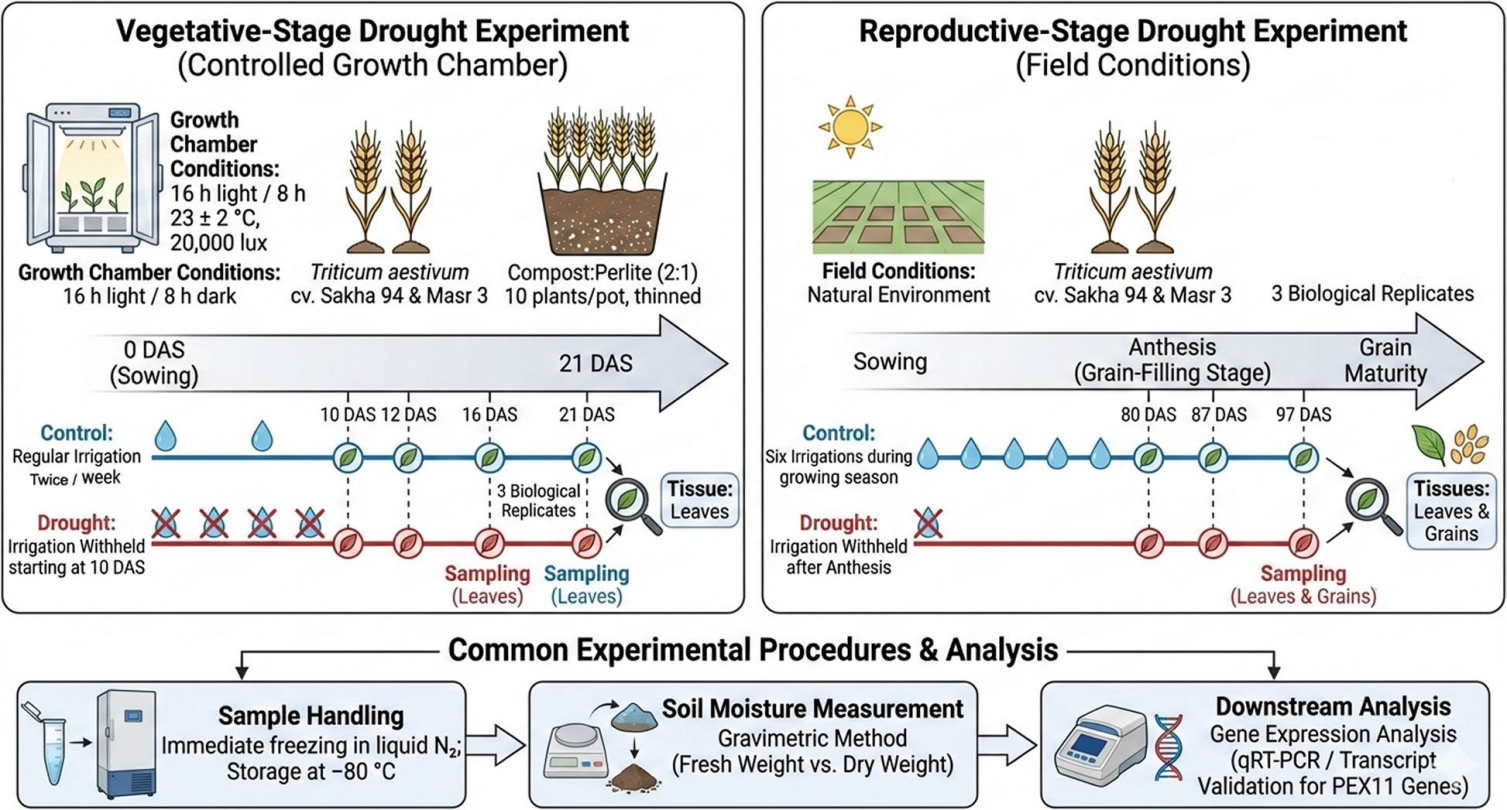
Schematic overview of vegetative- and reproductive-stage drought experiments used for *PEX11* expression analysis in wheat

To validate transcriptome-based predictions and examine the expression patterns of *PEX11* genes under drought stress, two complementary experiments were conducted using two bread wheat (*Triticum aestivum* L.) genotypes, Sakha 94 and Masr 3. The experiments were designed to assess drought responses at distinct developmental stages and under contrasting growth conditions: (i) a vegetative-stage drought experiment under controlled growth chamber conditions and (ii) a reproductive-stage drought experiment under field conditions. Figure 1. Schematic overview of vegetative- and reproductive-stage drought experiments used for *PEX11* expression analysis in wheat.

#### Vegetative-stage drought experiment under controlled conditions

Uniform seeds of Sakha 94 and Masr 3 were surface-sterilized using 1% (v/v) sodium hypochlorite for 10 min, thoroughly rinsed with sterile distilled water, and sown in plastic pots containing a compost:perlite mixture (2:1, v/v). Thirty seeds were initially sown per pot, and seedlings were later thinned to ten plants per pot to ensure uniform growth. Plants were grown in a controlled growth chamber under the following conditions: 16 h light/8 h dark photoperiod, light intensity of 20,000 lx, and temperature of 23 ± 2 °C. All pots were irrigated with tap water until 10 days after sowing (DAS), corresponding to the appearance of the second leaf. Plants were supplied with one-quarter-strength Hoagland nutrient solution every two weeks throughout the experiment. Drought stress was imposed by withholding irrigation from treatment pots starting at 10 DAS, while control plants continued to receive irrigation every two weeks. The experiment was conducted for a total duration of 21 DAS and arranged in a completely randomized design with three biological replicates per treatment. Leaves were taken after 10, 12, 16, 21 DAS, immediately frozen with liquid nitrogen and stored at −80 ºC until they were utilized to prepare for future analysis.

#### Reproductive-stage drought experiment under field conditions

A field experiment was conducted during the growing season using a split-plot design with three biological replicates. Each plot had a net area of 1.75 × 1.2 m, with manually sown rows spaced 15 cm apart. Standard agronomic practices were applied uniformly across all plots. Fertilizers were applied once at recommended rates of N:P:K (250:120:100 kg ha⁻^1^). Seedlings were thinned at 15 DAS to ensure uniform plant density. Control plots received a total of six irrigations throughout the growing season. Drought stress was imposed by withholding irrigation after anthesis, resulting in approximately half the number of irrigations compared to the control treatment. This strategy targeted drought stress during the grain-filling stage. Leaf and grain samples were collected from control and drought-stressed plants at 80, 87, and 97 DAS. All samples were immediately frozen in liquid nitrogen and stored at − 80 °C until further analysis. Soil moisture content was measured to confirm the effectiveness of drought stress imposition.

Soil samples were collected from control and drought-treated plots at the designated sampling time points. Fresh soil weight was recorded immediately after sampling, followed by oven drying at 105 °C until constant weight was achieved. Soil moisture content was calculated gravimetrically as: Soil moisture (%) = [(Fresh weight − Dry weight)/Dry weight] × 100. Quantitative soil moisture values for control and drought treatments at each sampling time point are provided in Supplementary Table S7. Soil water content was maintained near field capacity in control plots (25–28%) throughout the experiment, whereas drought-treated plots exhibited a progressive decline from approximately 16–17% at 80 DAS to below 10–11% at 97 DAS, confirming the effective imposition of drought stress across both cultivars.

### Biochemical measurements

Determination of H_2_O_2_ content, and catalase antioxidant enzyme were carried out for samples from both experiments. H_2_O_2_ was measured according to Yu et al. [21]. Catalase (CAT; EC 1.11.1.6) activity was assayed using the protein extract mentioned above. CAT activity was determined according to Hasanuzzaman and Fujita [22].

### Gene expression by of the *TaPEX11* genes qReal-time PCR

Total RNA was isolated from the samples using Triazole, Bioline, according to the instructions. The RNA samples were cleaned of genomic DNA contamination using DNase I from Thermo Scientific. Using the Sensifast 1 st cDNA synthesis kit, Bioline, two micrograms of RNA sample served as the template for the first strand cDNA synthesis. For the determination of relative gene expression levels of *TaPEX11s*, only seven representative *TaPEX11* genes were selected based on differential expression patterns in RNA-seq data under drought stress, subgenomic representation (A, B, and D genomes), and feasibility of specific primer design. Gene specific primers (Table S8) with SensiFast SYBR Lo-Rox (Bioline) 2X Master Mix were used. Specific primers were designed using Primer3 and checked for specificity for each template using Primer Blast. For that, the thermal cycling conditions were 95˚C for 5 min, followed by 40 cycles of 95 °C for 30 s, 57˚C for 30 s, and 72˚C f or 30 s in the STRATAGENE MxPro-3000P real-time PCR system. Expression of *TaPEX11* genes was normalized by *GAPDH* reference genes in both controls and treatments. All qRT-PCRs for each sample were done in three biological and three technical duplicates. Melting curve examination of the amplicons verified the specificity of the PCR reaction. The relative amounts of each transcript were determined using the comparative ΔΔCT method.

### Statistical analysis

All biochemical assays were performed using three independent biological replicates (*n* = 3) per treatment, unless otherwise stated. Data are presented as mean ± standard error (SE). All datasets met the assumptions required for parametric analysis. To determine whether there were significant differences between them, analysis of variance (ANOVA) was used at a significance level of *P* < 0.05. The least significant difference (LSD) test with a *P* < 0.05 was used to compare the treatments and identify any significant effects.

## Results

### *PEX11* homologous genes in wheat

*Arabidopsis* plant has five PEX11 proteins; AtPEX11A, AtPEX11B, AtPEX11C, AtPEX11D and AtPEX11E. A total of 12 *TaPEX11* genes have been identified. Due to alternative splicing of one gene (TraesCS4A02G442900), these genes encode 13 distinct protein isoforms. Supplementary Table S1 showing BlastP search results at EnsemblPlantswheat. The analysis of conserved domain revealed that wheat protein sequences of the PEX11 had a typical domain specific for peroxisomal biogenesis factor 11; pfam05648 (Supplementary Table S2, Figure S1, S2). Wheat has three genomes, (genome A, B and D). The identified PEX11 homologs are represented in the three genomes, five in genome A, four in genome B, and three in genome D. Remarkably, the 12 wheat *PEX11* genes are distributed in 9 chromosomes. Three of them are on Chr 2 A, 2B and 2D; six on Chr 4 A, 4B and 4D; one on each of Chr 5 A, Chr 7 A, and 7D. Interestingly, the deduced *TaPEX11* gene (TraesCS4A02G442900) shows alternative splicing, producing two transcript variants that encode the same protein with different amino acid length (Table 1).

**Table 1:** Description of the identified homologs of *PEX11* genes in the wheat genome

Transcript ID (IWGSC v1.2)	Gene name	Chr	Location Start–End	Strand	No. of introns	CDS (bp)	Protein length (AA)	MW (kDa)	pI	GRAVY	Instability index
TraesCS4D02G187900.1	*TaPEX11-1*	4D	326,980,467–326981474	reverse	0	1008	237	25.6	9.75	0.057	36.05
TraesCS4A02G117800.1	*TaPEX11-2*	4A	144,356,258–144,359,404	forward	0	1695	237	25.5	9.68	0.078	37.47
TraesCS4B02G186600.1	*TaPEX11-3*	4B	406,462,668–406465650	reverse	0	2983	237	25.5	9.84	0.049	36.59
TraesCS2D02G365100.1	*TaPEX11-4*	2D	470,210,955–470211644	reverse	0	690	229	25.4	9.68	−0.004	34.84
TraesCS2B02G385400.1	*TaPEX11-5*	2B	548,951,741–548,952,427	reverse	0	687	228	25.2	9.81	0.011	33.6
TraesCS2A02G368300.1	*TaPEX11-6*	2A	611,939,210–611941919	reverse	0	953	229	25.1	9.77	0.041	28.36
TraesCS4A02G442900.1	*TaPEX11-7.1*	4A	710,741,897–710744733	forward	6	1160	233	25.8	9.96	−0.02	25.87
TraesCS4A02G442900.2	*TaPEX11-7.2*	4A	710,741,897–710744733	forward	5	1079	206	22.7	10.13	−0.035	21.51
TraesCS4B02G355100.1	*TaPEX11-8*	4B	646,193,428–646,195,589	forward	6	1411	237	26.2	9.93	−0.034	25.07
TraesCS4D02G348600.1	*TaPEX11-9*	4D	502,072,761–502074897	forward	6	1120	237	26.2	9.98	−0.045	26.99
TraesCS7A02G056300.1	*TaPEX11-10*	7A	27,095,949–27098638	forward	6	1192	233	25.8	9.91	0.003	22.73
TraesCS7D02G051200.1	*TaPEX11-11*	7D	26,720,429–26721737	forward	6	702	233	25.8	9.96	−0.013	23.09
TraesCS5A02G524400.1	TaPEX11-12	5A	685,004,226–685006607	forward	6	1128	237	26.3	9.98	−0.023	24.43

Amino acids of wheat PEX11 proteins were analyzed and all proteins parameters are listed in Table 1. The length of TaPEX11 proteins varied from 206 to 237 amino acids. EXPASY analysis predicted very close isoelectric point (pI) values for all TaPex11 protein sequences (ranging from 9.68 to 10.13). And molecular weight (ranging from 22.7 kDa to 26.3 kDa). All proteins are predicted to be stable as they have stability index < 40.

**Fig. 2:**
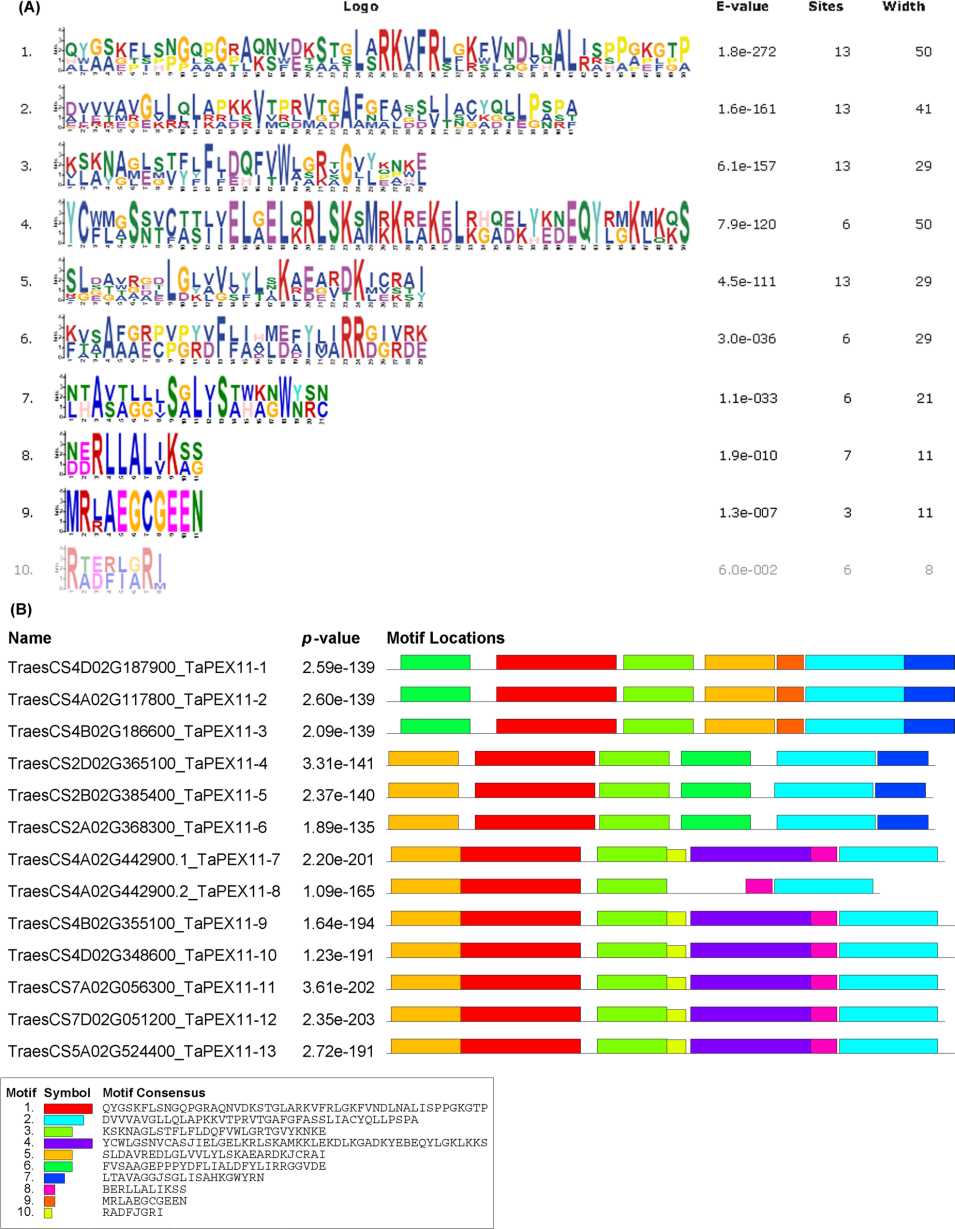
Conserved motif distribution in protein sequences of *PEX11* predicted homologs in wheat. The MEME (Multiple Expectation Maximization for Motif Elicitation) model predicts the distributions of conserved motifs (**A**) and *TaPEX11* protein motifs (**B**). Different colored boxes with matching numbers reflect the motifs. Table S3 contains a list of the motif descriptions

Motif analysis revealed the presence of 10 distinct motifs across the proteins. Figure (2A) represents the sequence logos for the 10 identified motifs in PEX11 homolog proteins from wheat, as identified by the MEME suite. These logos provide a visual representation of the sequence conservation and variability within the motifs, along with statistical data such as e-values, the number of sites, and motif width. Each logo illustrates the sequence conservation for each motif, where larger letters indicate higher conservation at specific positions. Motifs 1, 4, and 9 exhibit the highest sequence conservation, as evidenced by the dominance of certain amino acids across their positions while the other motifs displayed more variability. Distribution and occurrence of motifs are shown in Fig. (2B). Motif 1, Motif 2, Motif 3, and Motif 5 were the most prominent, appearing in all 13 homologs. Distribution and occurrence of motifs along the protein sequences classified the family into three groups; each group contains the conserved motifs at the corresponding position (group I from PEX11-1: PEX11-3), (group II from PEX11-4: PEX11-6) and (group III from PEX11-7.1: PEX11-12) (Fig. 2). The motifs identified were highly significant, with *p*-values ranging from 2. 5 × 10^−139^ to 2.7 × 10^−191^, underscoring the reliability of the identified conserved regions. Supplementary Table S3 shows the sequence, length and identification of the ten motifs. Motifs 1, 2 and 4 were identified as peroxisomal biogenesis factor 11 specific motifs by motif scan database. Although that Motif 3 and 5 were found in all identified TaPEX11 sequences, they were not defined by motif databases, indicating that these motifs are previously unannotated regions which suggest being novel motifs for peroxisomal biogenesis factor domains.

### TaPEX11 3D structure models

**Fig. 3:**
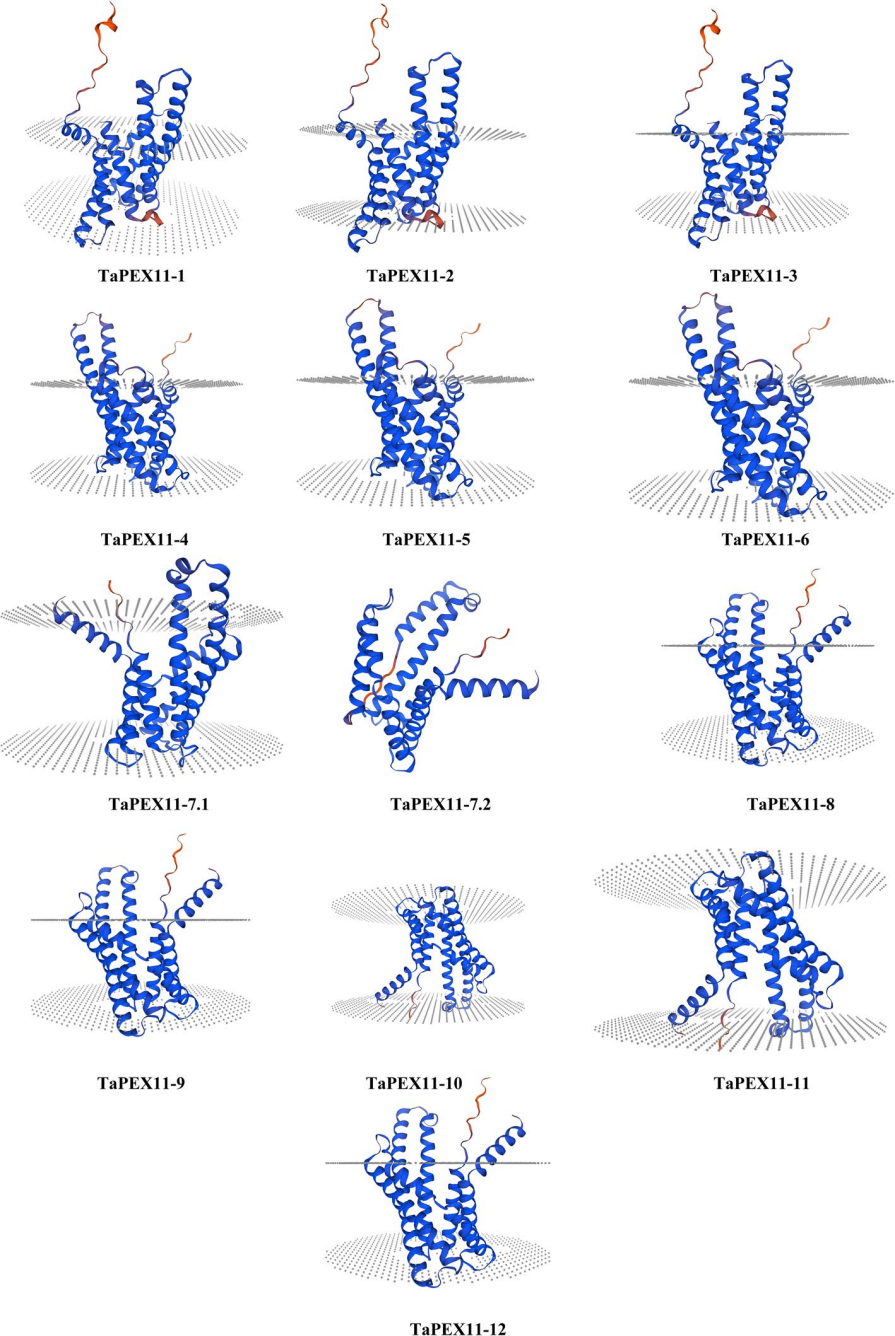
Predicted 3D structures of peroxin 11 (PEX11) proteins in wheat (TaPEX11-1 to TaPEX11-12). The ribbon representation was chosen to highlight the overall topographical progression of the polypeptide chain, rather than atomic details such as hydrogen bonding (coloured blue and red along the chain, N-term to C-term; helical ribbons are α-helices, straight ribbons are β-strands; each structure in front and back view, related by 180-degree rotation)

The 3D structural models of 13 putative PEX11 proteins of wheat (TaPEX11-1 to TaPEX11-12, and isoforms 7.1 and 7.2) are shown in Fig. 3. The selected template identifiers, percent sequence identity, alignment coverage, GMQE scores are summarized in Supplementary Table S4. The models of all the proteins are mainly made up of transmembrane domains, which is in accordance with the established function of PEX11 proteins in peroxisomal membrane dynamics. The alpha-helical regions, which are colored blue, form the core structure, and the B-sheets colored red. Variations in the spatial organization of helices and loop regions reflect structural diversity among the TaPEX11 isoforms. The orientation within the membrane is consistent across the models except for TaPEX11-7.2 as the transmembrane segment couldn’t be identified. The quality of models was confirmed by the Ramachandran plot presented in Figure S3 evaluates the stereochemical quality of the predicted 3D structure for the protein model. Most of the residues are concentrated in the most favoured regions (dark green) and allowed regions (light green) of the plot, which shows that the backbone dihedral angles (Φ and Ψ) of the protein are in conformationally favourable states. This suggests a good quality model with minimal steric clashes or structural errors. There are not many residues in less favoured regions, which could be equivalent to flexible or functionally important regions such as termini or loops. Overall, the distribution of residues confirms the validity and structural integrity of the predicted 3D model. However, consistent with the intrinsic challenges of modelling membrane proteins, these structures should be interpreted as approximate representations of overall fold and transmembrane organization rather than high-resolution atomic models.

### Phylogenetic analyses of TaPEX11 proteins

**Fig. 4:**
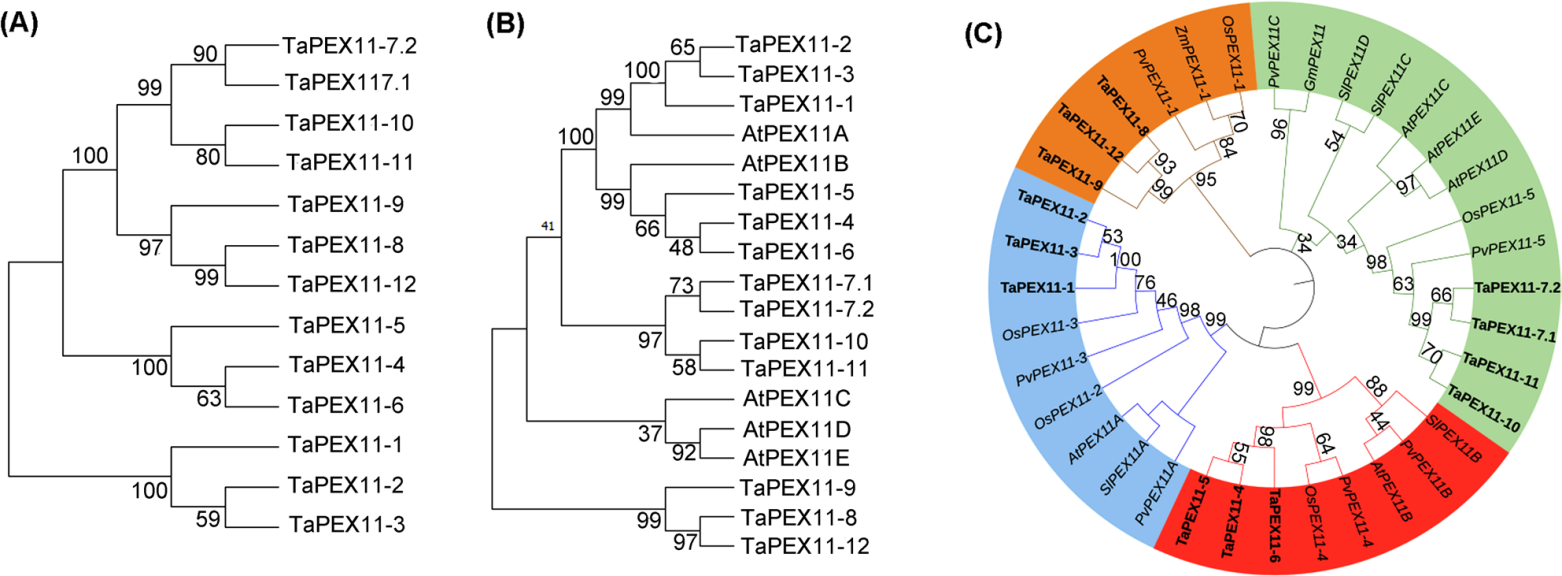
Phylogenetic analysis of PEX11 proteins in wheat and across plant species. The tree was constructed using the Maximum likelihood method using MEGA 11 software with 1,000 bootstrap replicates; bootstrap values are shown at branch nodes. **A** PEX11 tree in wheat highlighting lineage-specific duplication, **B** PEX11 tree in wheat and model plant *Arabidopsis* illustrating orthologous relationships, **C** PEX11 tree in different monocot and dicot species showing major evolutionary clades. Different clades are marked with distinct colors, showing evolutionary relationships and potential functional diversification among PEX11 proteins across species. TaPEX11: PEX11 proteins in wheat, ZmPEX11: PEX11 proteins in maize, OsPEX11: PEX11 proteins in rice, PvPEX11: PEX11 proteins in beans, SlPEX11: PEX11 proteins in tomato, AtPEX11: PEX11 proteins in *Arabidopsis*. Supplement Table S5 shows scientific names and gene identifiers of these sequences

Multiple sequence alignment of TaPEX11 proteins revealed the conserved and divergent regions within the sequences (Figure S4). Highly conserved domains were observed throughout the alignment. A phylogenetic tree was generated to resolve the evolutionary relationships among PEX11 proteins from different plant species. The phylogenetic tree (Fig. 4A) shows the relationship of TaPEX11 proteins in *T. aestivum* and reveals four well-supported clades (bootstrap values > 90%), reflecting lineage-specific gene duplication events consistent with the polyploid nature of *T. aestivum*. Members within each clade exhibit high sequence similarity, suggesting functional conservation among paralogs. These clades correspond closely with conserved motif compositions (Fig. 2), supporting the hypothesis that evolutionary divergence among PEX11 subfamilies is accompanied by functional specialization rather than random sequence drift. Members of clade 1 (TaPEX11-1, TaPEX11-2, and TaPEX11-3) shared an identical distribution and composition of conserved motifs across their protein sequences (Fig. 1). Similarly, TaPEX11-4, TaPEX11-5, and TaPEX11-6 clustered in clade 2 and exhibited a distinct but internally conserved motif arrangement. Proteins belonging to clades 3 and 4 also displayed consistent motif distributions within each clade, indicating that phylogenetic grouping closely corresponds to conserved sequence features. Gene structure analysis further supported this clade-specific organization (Fig. 4A). Genes in clades 1 and 2 were intronless, whereas genes belonging to clades 3 and 4 contained multiple introns. This clear structural distinction among clades suggests differential evolutionary constraints and supports the functional relevance of the phylogenetic classification. To assess orthologous relationships with a model dicot species, Fig. 4B integrates the PEX11 in wheat and *Arabidopsis*. The tree divided into five clades. AtPEX11A in the first group with TaPEX11-1, TaPEX11-2, and TaPEX11-3. AtPEX11B in the second group with TaPEX11-4, TaPEX11-5, and TaPEX11-6. Whereas AtPEX11C, AtPEX11D, and AtPEX11E were in separate clade, suggesting lineage-specific diversification. Figure 4D presents a comprehensive phylogenetic analysis across monocot and dicot species, clearly separating PEX11 proteins into four main clades, which are marked in various colors for simplicity. In general, the phylogenetic analysis indicates that PEX11 proteins have experienced lineage-specific diversification with conserved functional clusters.

### Gene structure and chromosomal location of *TaPEX11* genes

**Fig. 5:**
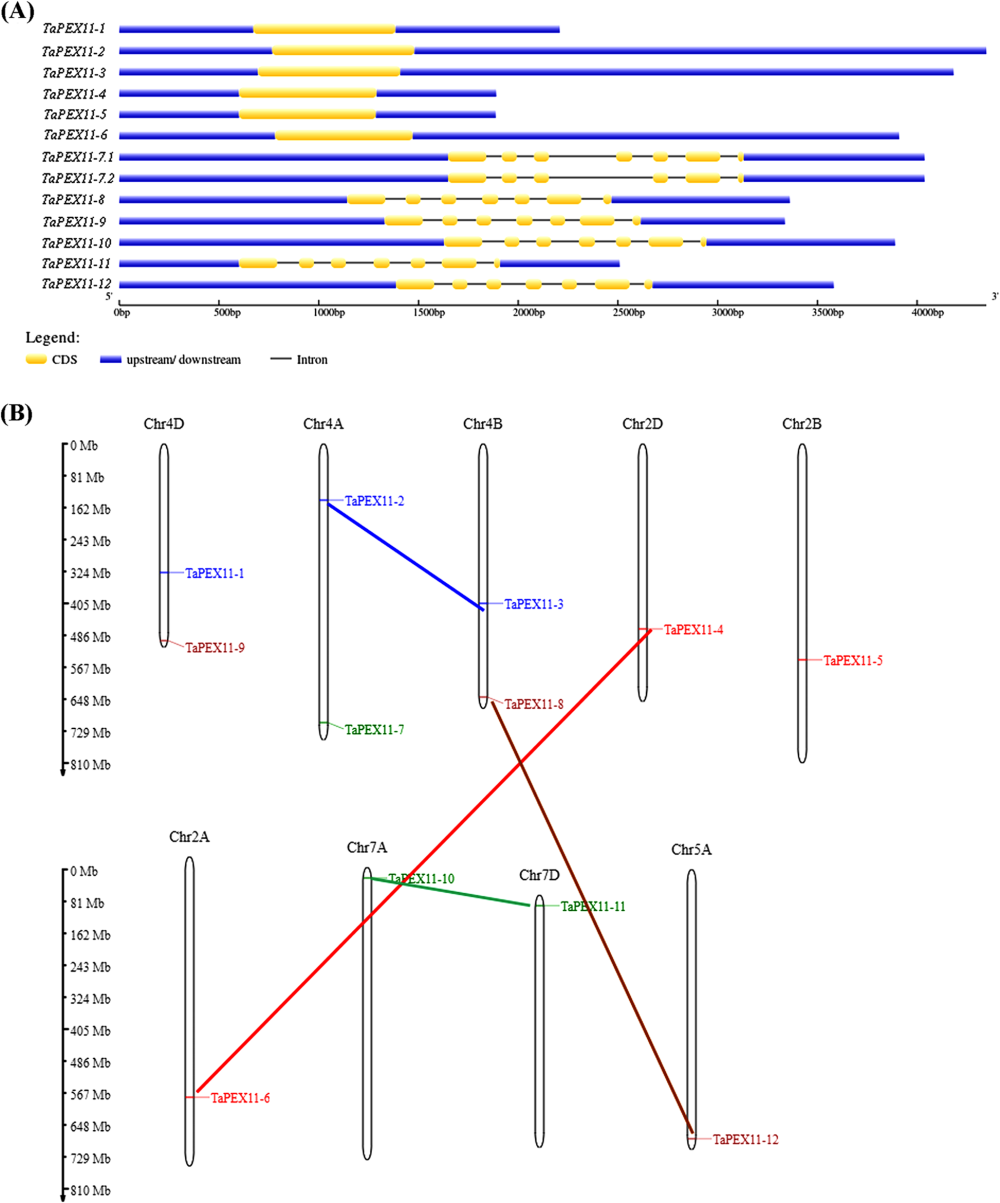
Genomic analysis of *PEX11* paralogous genes in wheat. **A** Gene structure analysis of *PEX11* genes in wheat. The exons are shown as blue boxes interspaced by introns shown as a thin black line. **B** Genomic positions of *PEX11* genes on wheat chromosomes of A, B and D sub-genomes. Chromosome names are indicated at the top of each bar (Chr4D, Chr4A, Chr4D, Chr2D, Chr2A, Chr2B, Chr7D, Chr7A, and Chr5A). Duplicated chromosomal segments are connected by line connectors. The scale on the left is in megabases. Different colours represent the same colours of clades’ members in Fig. 3

The Gene Structure Display Server was used to study the gene structure of 13 predicted *PEX11* genes in wheat. There was variation within the exon–intron organization by different gene lengths, reflecting evolution but also the functional diversity in the *PEX11* gene family. The number of exons across the PEX11 homologs varied, some genes contained multiple exons (e.g., *TaPEX11-8*, *TaPEX11-9***,** *TaPEX11-10*, *TaPEX11-11*, and *TaPEX11-12*), while splice variants of *TaPEX11-7* exhibited fewer exons, and the first six homologs were only one exon (Fig. 5A). Introns were present between exons and showed variability in their lengths, with some genes having longer introns (e.g., splice variants of *TaPEX11-7*; TraesCS4A02G442900) and others shorter ones. Blue bars indicate upstream and downstream regions, delineating UTRs and potential regulatory elements.

Chromosomal mapping demonstrated that the twelve wheat *PEX11* genes were scattered on chromosomes 4D, 4 A, 4B, 2D, 2 A, 2B, 7 A, 7D, and 5 A, with one *PEX11* gene localized to each of 2D, 2 A, and 2B, 7 A, 7D and 5 A, and a pair of *PEX11* genes found at 4D, 4 A and 4D (Fig. 5B). Furthermore, *TaPEX11-8*, *TaPEX11-9*, *TaPEX11-10*, *TaPEX11-11*, and *TaPEX11-12* are positioned near the chromosome ends. Meanwhile, some underwent a parallel duplication process where genes were doubled. They were (*TaPEX11-2*-*TaPEX11-3*), (*TaPEX11-4*-*TaPEX11-6*), (*TaPEX11-8*-*TaPEX11-12*), and (*TaPEX11-10*-*TaPEX11-11*) genes.

### Ka/Ks analysis, gene duplication, and collinearity of *TaPEX11* within wheat genome and other species

**Fig. 6:**
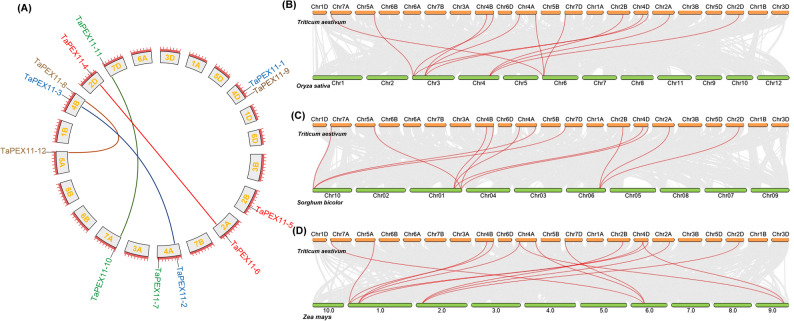
Gene duplication and collinearity of *TaPEX11* within wheat genome and other species. **A** Circos plot illustrating the chromosomal distribution and syntenic relationships of *PEX11* genes in wheat. The outermost circle represents the wheat sub-genomes chromosomes. The positions of *PEX11* genes are marked with labels. Segmental duplication between *PEX11* paralogs on Chr2A and Chr2D, Chr4A and Chr4B, Chr7A and Chr7B, and Chr4B and Chr5A Chr1 is represented by connecting lines. **B** Syntenic relationships between *Triticum aestivum* chromosomes (orange bars) and *Oryza sativa* chromosomes (green bars, Chr01–Chr12), **C** Syntenic relationships between *Triticum aestivum* chromosomes (orange bars) and *Sorghum bicolor* (green bars, Chr01–Chr10), **D** Syntenic relationships between *Triticum aestivum* chromosomes (orange bars) and *Zea mays* chromosomes (green bars, Chromosomes 1.0–10.0). Syntenic relationships are shown with connecting arcs. Grey arcs indicate conserved syntenic blocks across the genomes and red arcs highlight positions of conserved *PEX11* genes between wheat and other species

The different types of selection measured by calculating the non-synonymous (Ka) to synonymous (Ks) substitution ratio (Ka/Ks) at the population genetics level. Ka/Ks = 1 is interpreted as neutral selection, Ka/Ks < 1 as purifying selection, and Ka/Ks > 1 as positive selection. Gene identification for *TaPEX11-2*, *TaPEX11-3*, *TaPEX11-4*, *TaPEX11-6*, *TaPEX11-8*, *TaPEX11-12*, *TaPEX11-10*, and *TaPEX11-11* was carried out through BLASTp analysis. KaKs calculator in TBtools software was used to calculate the Ka/Ks values of orthologous typical *PEX11* genes in wheat to detect molecular selection effects. The results revealed that the Ka/Ks values between these duplicated gene pairs were less than 1, indicating that *TaPEX11* genes were primarily subject to purifying selection during the evolutionary process within *T. aestivum* (Table 2). According to the analysis results of the MCScanX program package and based on the sequence similarity and chromosome position, a total of four pairs of segmental duplications were found among 12 *TaPEX11*, while no tandem duplication event was identified (Fig. 6A). Chromosome 4B had two segmental duplications of *TaPEX11* genes with chromosomes 4 A and 5 A, while additional duplicated gene pairs were detected between chromosomes 7 A and 7D and between chromosomes 4 A and 7D. These duplication patterns reflect both homoeologous relationships derived from wheat polyploidization and segmental duplication events occurring after genome merger. Collectively, these results suggest that the expansion and chromosomal distribution of the *TaPEX11* gene family were shaped by a combination of whole-genome duplication associated with wheat polyploid evolution, followed by segmental duplications and chromosomal rearrangements.

**Table 2:** Ka/Ks between wheat paralogous *PEX11* pairs

**Paralogous Pair**	**Ka**	**Ks**	**Ka/Ks**	**T (MYA)**
TraesCS4A02G117800 (*TaPEX11-2*)—TraesCS4B02G186600 (*TaPEX11-3*)	0.013568453	0.048819761	0.27792952	3.755366235
TraesCS2D02G365100 (*TaPEX11-4*)—TraesCS2A02G368300 (*TaPEX11-6*)	0.03044575	0.120135323	0.253428796	9.241178707
TraesCS4B02G355100 (*TaPEX11-8*)—TraesCS5A02G524400 (*TaPEX11-12*)	0.011758093	0.092244766	0.127466231	7.095751201
TraesCS7A02G056300 (*TaPEX11-10*)—TraesCS7D02G051200 (*TaPEX11-11*)	0.001910221	0.158650067	0.012040466	12.20385127

To uncover evolutionary insights into the *TaPEX11*, two genome-wide comparative syntenic maps were created for wheat (*Triticum aestivum* L.) and rice (*Oryza sativa* L.), sorghum (*Sorghum bicolor*), and maize (*Zea mays* L.) (Fig. 6B, C, D). The results revealed 12 orthologous gene pairs between rice and with 12 *PEX11* genes in wheat (Fig. 6B, Table 3), 12 genes in sorghum had orthologous relationships with 12 *PEX11* genes in wheat (Fig. 6C, Table 3), and 14 genes in maize had orthologous relationships with 12 *PEX11* genes in wheat (Fig. 6D, Table 3). These results revealed a strong synteny relationship between species.

**Table 3:** The colinear *PEX11* gene pairs in wheat and rice, sorghum, or maize

*T. aestivum* Chr	Gene ID	*O. sativa* Chr	Gene ID
Chr7A	TraesCS7A02G056300 (*TaPEX11-10*)	Chr6	LOC_Os06g03660
Chr5A	TraesCS5A02G524400 (*TaPEX11-12*)	Chr3	LOC_Os03g02590
Chr4B	TraesCS4B02G186600 (*TaPEX11-3*)	Chr3	LOC_Os03g19000
Chr4B	TraesCS4B02G355100 (*TaPEX11-8*)	Chr3	LOC_Os03g02590
Chr4A	TraesCS4A02G117800 (*TaPEX11-2*)	Chr3	LOC_Os03g19000
Chr4A	TraesCS4A02G442900 (*TaPEX11-7*)	Chr6	LOC_Os06g03660
Chr7D	TraesCS7D02G051200 (*TaPEX11-11*)	Chr6	LOC_Os06g03660
Chr2B	TraesCS2B02G385400 (*TaPEX11-5*)	Chr4	LOC_Os04g45210
Chr4D	TraesCS4D02G187900 (*TaPEX11-1*)	Chr3	LOC_Os03g19000
Chr4D	TraesCS4D02G348600 (*TaPEX11-9*)	Chr3	LOC_Os03g02590
Chr2A	TraesCS2A02G368300 (*TaPEX11-6*)	Chr4	LOC_Os04g45210
Chr2D	TraesCS2D02G365100 (*TaPEX11-4*)	Chr4	LOC_Os04g45210

***T. aestivum* Chr**	**Gene ID**	***S. bicolor* Chr**	**Gene ID**
Chr7A	TraesCS7A02G056300 (*TaPEX11-10*)	Chr10	Sobic.010G016400
Chr5A	TraesCS5A02G524400 (*TaPEX11-12*)	Chr01	Sobic.001G531500
Chr4B	TraesCS4B02G186600 (*TaPEX11-3*)	Chr01	Sobic.001G399100
Chr4B	TraesCS4B02G355100 (*TaPEX11-8*)	Chr01	Sobic.001G531500
Chr4A	TraesCS4A02G117800 (*TaPEX11-2*)	Chr01	Sobic.001G399100
Chr4A	TraesCS4A02G442900 (*TaPEX11-7*)	Chr10	Sobic.010G016400
Chr7D	TraesCS7D02G051200 (*TaPEX11-11*)	Chr10	Sobic.010G016400
Chr2B	TraesCS2B02G385400 (*TaPEX11-5*)	Chr06	Sobic.006G160400
Chr4D	TraesCS4D02G187900 (*TaPEX11-1*)	Chr01	Sobic.001G399100
Chr4D	TraesCS4D02G348600 (*TaPEX11-9*)	Chr01	Sobic.001G531500
Chr2A	TraesCS2A02G368300 (*TaPEX11-6*)	Chr06	Sobic.006G160400
Chr2D	TraesCS2D02G365100 (*TaPEX11-4*)	Chr06	Sobic.006G160400

***T. aestivum* Chr**	**Gene ID**	***Z. mays* Chr**	**Gene ID**
Chr7A	TraesCS7A02G056300 (*TaPEX11-10*)	6	Zm00001d036001_T006
Chr5A	TraesCS5A02G524400 (*TaPEX11-12*)	1	Zm00001d027366_T001
Chr4B	TraesCS4B02G186600 (*TaPEX11-3*)	1	Zm00001d028834_T001
Chr4B	TraesCS4B02G355100 (*TaPEX11-8*)	1	Zm00001d027366_T001
Chr4A	TraesCS4A02G117800 (*TaPEX11-2*)	1	Zm00001d028834_T001
Chr4A	TraesCS4A02G442900 (*TaPEX11-7*)	6	Zm00001d036001_T006
Chr4A	TraesCS4A02G117800 (*TaPEX11-2*)	9	Zm00001d047676_T002
Chr7D	TraesCS7D02G051200 (*TaPEX11-11*)	6	Zm00001d036001_T006
Chr2B	TraesCS2B02G385400 (*TaPEX11-5*)	2	Zm00001d002834_T001
Chr4D	TraesCS4D02G187900 (*TaPEX11-1*)	1	Zm00001d028834_T001
Chr4D	TraesCS4D02G348600 (*TaPEX11-9*)	1	Zm00001d027366_T001
Chr4D	TraesCS4D02G187900 (*TaPEX11-1*)	9	Zm00001d047676_T002
Chr2A	TraesCS2A02G368300 (*TaPEX11-6*)	2	Zm00001d002834_T001
Chr2D	TraesCS2D02G365100 (*TaPEX11-4*)	2	Zm00001d002834_T001

### Distribution of *Cis*-regulatory elements (CREs) in the promoters of *TaPEX11* genes

**Fig. 7:**
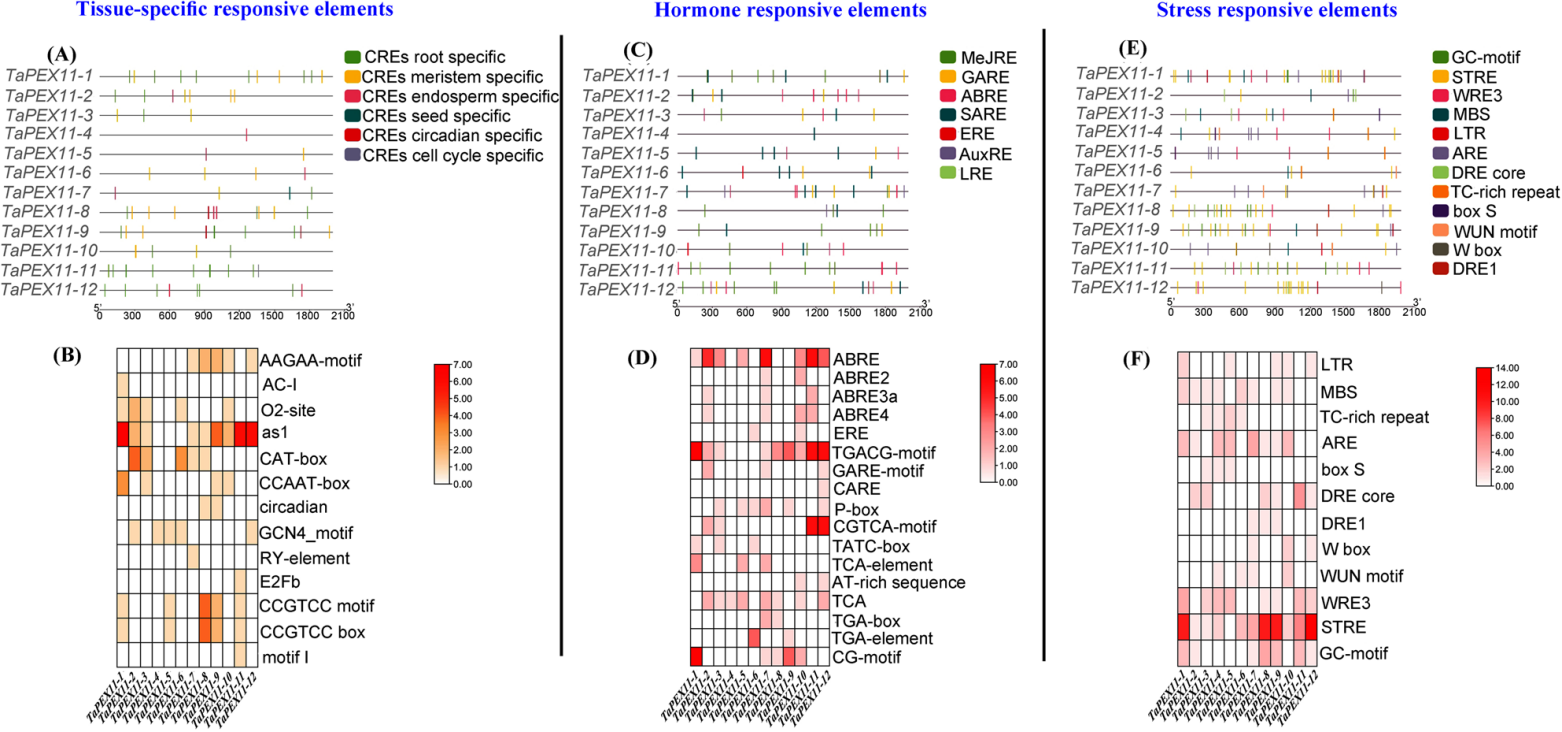
*Cis-*regulatory elements in the wheat *PEX11* gene promoter region. **A** Location of the tissue-specific responsive elements. **B** Frequency of tissue-specific responsive elements of the *TaPEX11* promoters. **C** Location of the hormone responsive elements. **D** Frequency of hormone related elements of the *TaPEX11* promoters. **E** Location of the stress responsive elements. **F** Frequency of stress responsive elements of the *TaPEX11* promoters. The detailed number of *cis*-element in each category for each *TaPEX11* gene is provided in Table 4 and Table S9

Wheat *PEX11* gene promoter regions were analyzed for *cis*-regulatory elements (CREs), and the results showed that there are different functional elements potentially involved in tissue-specific expression, hormone and stress regulation (Fig. 7). Distribution of *cis*-elements of the promoter regions of *PEX11* homologous genes are represented in (Table S9). There is a different distribution of such elements among *Pex11* gene promoters; thus, they could contribute to the regulation of gene activity under various conditions. Promoter and site binding elements were the most abundant, hormone-related elements (included ABA, auxin, gibberellins, and salicylic acid responsive elements), stress-responsive elements (especially those for drought, salinity, and heat stresses), and the tissue-specific elements were represented in all promoters with various abundance. Notably, tissue-specific and hormone related elements fairly represented in *TaPEX11-4* promoter (Table 4).

**Table 4:** Number of cis-regulatory element (CRE) categories identified in the 2-kb upstream promoter regions of wheat *TaPEX11* genes

Wheat *PEX11* genes	Light responsive elements	Hormone responsive elements	Stress responsive elements	Tissue specific elements	Promoter and site binding elements	Other elements
*TaPEX11-1*	9	19	24	14	32	46
*TaPEX11-2*	9	15	6	9	42	30
*TaPEX11-3*	10	8	8	5	75	15
*TaPEX11-4*	11	1	12	1	110	12
*TaPEX11-5*	12	7	10	3	80	17
*TaPEX11-6*	13	7	7	5	55	7
*TaPEX11-7*	19	20	13	4	67	21
*TaPEX11-8*	3	6	19	13	50	29
*TaPEX11-9*	4	10	19	12	44	33
*TaPEX11-10*	11	14	11	5	68	6
*TaPEX11-11*	14	22	17	10	47	50
*TaPEX11-12*	10	22	19	8	60	41

*TaPEX11* genes generally had root-specific, meristem-specific, endosperm-specific, and seed-specific elements dispersed across them, with some genes displaying relatively higher densities of these regulatory motifs (Fig. 7A). Genes have elements in the order of abundance include *TaPEX11-3*, *TaPEX11-4*, *TaPEX11-5*, *TaPEX11-6*, *TaPEX11-7*, and *TaPEX11-10*. Interestingly, *TaPEX11-4* possessed endosperm-specific elements only, while *TaPEX11-5* contained endosperm- and meristem-specific elements. The *TaPEX11-7* and *TaPEX11-8* had specific circadian elements which were suspected to involve possible diurnal regulation of gene expression. The most prevalent tissue-specific element was as1 in *TaPEX11-1*, *TaPEX11-11*, and *TaPEX11-12* (Fig. 7B). A number of involved hormones-responsive elements have been detected in the promoter regions of *TaPEX11* (Fig. 7C). These are MeJA-responsive elements (MeJREs), gibberellin-responsive elements (GARES), abscisic acid-responsive elements (ABRES), SAREs (salicylic acid-responsive elements), ERE (ethylene-responsive elements), and AuxREs (auxin-responsive elements). The presence of such elements suggests that *TaPEX11* genes might be under the regulation of different plant hormones, contributing to the genes' functions in developmental and stress-related processes. Hormone-responsive elements (Fig. 7D) have variable frequencies of ABRE, TGACG-motif, and CGTCA-motif; this suggests differences in hormone regulation among *TaPEX11* members. The most interesting observation is SARE's presence only in *TaPEX11-4*. Besides, stress-responsive elements were abundant in all *TaPEX11* promoter regions (Fig. 7E). Elements involved in responses to drought stress (DRE, MBS), oxidative stress (W box, ARE), and temperature stress (STRE), were found of the *TaPEX11* genes. Also, WUN motifs, implicated in reaction to wounding, were detected in some promoter regions. Stress-responsive elements (Fig. 7F) also show a high frequency of STRE (stress response) motifs in some of the genes (*TaPEX11-1*, *TaPEX11-8*, *TaPEX11-9*, and *TaPEX11-12*), hence, strengthen the claim that they play a role in abiotic stress tolerance.

### Co-expression networking and transcription factors

**Fig. 8:**
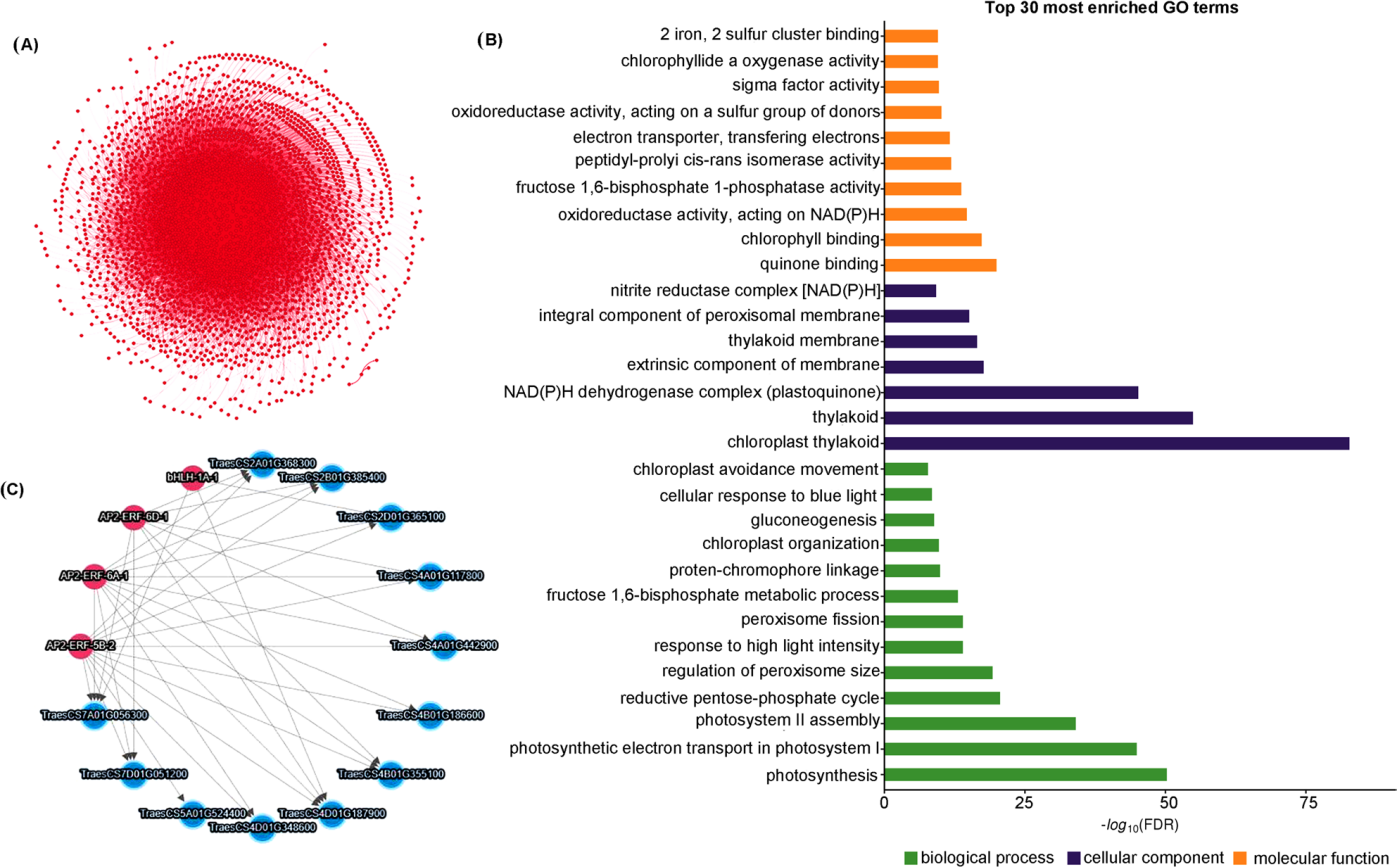
Network analysis of *PEX11* genes and their co-expressed genes and transcription factor interaction. **A** Gene co-expression network reconstructed utilizing transcriptomic data, demonstrating the global interaction landscape among all analyzed genes. Node means gene, while edge indicates a significant co-expression relationship, thus revealing a potential co-regulatory or functional association. **B** Gene Ontology (GO) enrichment analysis of co-expressed genes. For the 30 significantly enriched GO terms, all top three categories are biological process (green), cellular component (purple), and molecular function (orange). It is then expressed in terms of enrichment significance as—log10 (FDR). Such key enriched terms would include "photosynthesis", "chloroplast thylakoid", and "oxidoreductase activity" inferring that candidate gene networks have a role in photosynthetic regulation and redox processes especially under the stress conditions. **C** The transcription factor (TF) gene interactions of the main candidate genes. Transcription factors are represented as pink nodes and *TaPEX11* genes as blue nodes. The lines represent regulatory interactions primarily founded on co-expression and TF binding information. Prominent among those include members of the AP2/ERF family (like the AP2-ERF-6D4, AP2-ERF-6A4, and AP2-ERF-5B-2) and bHLH-14–4, marking them as key regulators implied in the expression control of stress-responsive genes

Gene co-expression networks used to identify important *PEX11* co-expressed genes in wheat multi tissues and the regulatory roles of transcription factors (TFs) genes further investigated by the interaction between TFs and *PEX11* target genes (Fig. 8).

### Co-expression networking enrichment analysis of *TaPEX11* genes

The complex network of *PEX11* genes and their co-expressed patterns control different biological and physiological mechanisms in the plant and is therefore important for plant responses to different environmental conditions. A co-expression network was constructed for *TaPEX11s* based on a Pearson’s correlation coefficient (PCC) threshold of 0.9 of the expression data of PRJEB25639, and we identified 1299 genes in wheat multiple tissues that are involved in the network together with the *TaPEX11s* (Fig. 8A; Table S10). Six *TaPEX11s* genes were included in the co-expression network with other 1293 wheat genes that may be participating in different regulatory mechanisms. Gene ontology (GO) enrichment analyses classified the co-expressed genes into three types of GO classes: 1) biological process (101); 2) cellular component (15); and 3) molecular function (67) with enrichments of < 0.05 in p-values. The detailed list of GO description, statistics, and corresponding genes is given in Table S11. Importantly, several enriched GO terms were directly related to peroxisome biology and stress adaptation, including regulation of peroxisome size (GO:0044375), peroxisome fission, response to high light intensity (GO:0009644), and redox-related oxidoreductase activity (Fig. 8B). In addition, GO terms associated with photosynthesis, photosynthetic electron transport, chloroplast organization (GO:0009658), and the reductive pentose-phosphate cycle were significantly enriched. Although these processes are not peroxisomal, they are functionally linked to peroxisomes through photorespiration, ROS metabolism, and metabolic crosstalk between chloroplasts and peroxisomes, particularly under stress conditions. The enrichment of gluconeogenesis (GO:0006094) further supports a role for *TaPEX11*-associated networks in coordinating carbon metabolism during stress-induced metabolic reprogramming. Overall, the co-expression patterns suggest that *TaPEX11* genes play a central role in regulating peroxisome dynamics and associated stress-responsive metabolic pathways, thereby contributing to plant homeostasis under fluctuating environmental conditions.

### Transcription factors enrichment and regulatory analysis of *TaPEX11* genes

To ascertain the possible transcriptional regulation of *PEX11* genes in wheat, TF enrichment analysis was implemented. Out of the predicted regulators, four transcription factors significantly enriched (Table 5; Fig. 8C). The strongest association was with the AP2-ERF family. AP2-ERF-5B-2 occurred in all predicted targets with 100% occurrence, significant at *p*-value of 7.73 × 10⁻^4^ and was moderate with fold change of 1.92. AP2-ERF-6A-1 was found in 91.67% of the targets with *p* = 1.98 × 10⁻^3^; fold change = 2.06; AP2-ERF-6D-1 associated with 66.67% of the genes with *p* = 1.11 × 10⁻^2^; fold change = 2.50. Although bHLH-1A-1 was found in only 25% of the predicted regulatory interactions (TraesCS2D01G365100, TraesCS4D01G187900, TraesCS7A01G056300), it had the largest fold change (10.19) lending credence to the possibility that it has a strong but specific regulatory effect (*p* = 2.86 × 10⁻^2^). The predicted targets of each TF are shown in Table S12. These results demonstrate considerable involvement of the AP2-ERF transcription factors in regulating the expression of *PEX11* genes in wheat, with bHLH likely having a significant role.

**Table 5:** Transcription factors enrichment analysis for prediction of transcription regulation of *PEX11* genes in wheat

TF	Count	Ratio (%)	*P* value	Fold Change
AP2-ERF-5B-2	12	100.00	7.73e-4	1.92
AP2-ERF-6A-1	11	91.67	1.98e-3	2.06
AP2-ERF-6D-1	8	66.67	1.11e-2	2.50
bHLH-1A-1	3	25.00	2.86e-2	10.19

### Expression profiles of *TaPEX11* genes

**Fig. 9:**
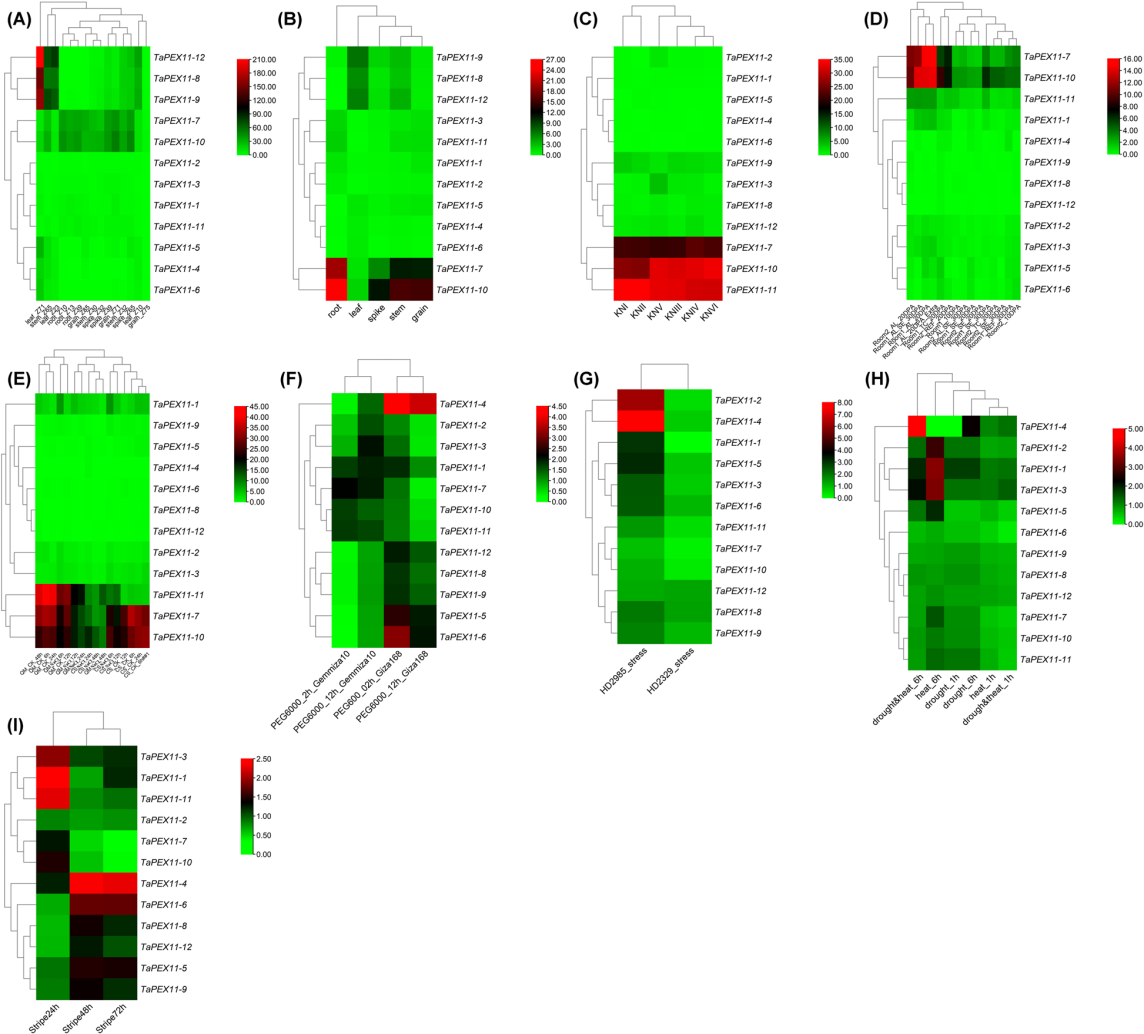
Heatmaps representing the expression profiles of *TaPEX11* homologous genes in wheat under various developmental stages, tissues, and stress conditions based on publicly available RNA-seq datasets. **A** Expression across different developmental stages, **B** Expression in various wheat tissues, **C** Expression at early spike development, **D** Expression during grain development stages, **E** Expression under salinity stress, **F** Expression in different genotypes (Giza168 and Gmiza10) under PEG600 for 2 h and 12 h, **G** Expression in heat tolerant (HD2985) and sensitive (HD2329) genotypes, **H** Expression under drought, heat, and combined stress conditions for 1 h and 6 h, **I** Expression under strip rust infection for 24, 48, and 72 h. Gene expression values were normalized and log2-transformed. log₂TPM values were normalized within each dataset and subsequently scaled across comparable samples using quantile normalization to ensure consistency of expression patterns across conditions. Red indicates high expression, black indicates moderate expression, and green indicates low expression. Hierarchical clustering was performed for both genes and samples

The analyses of gene spatiotemporal expression specificity could provide valuable information for studying the function of *PEX11* genes in wheat growth and development. The RNA-seq data were downloaded to analyze the spatial and temporal expression profiles of typical *TaPEX11* genes in wheat. Moreover, the spatiotemporal-specific expression clustering heatmap was plotted based on log2 TPM (transcripts per million) values (Fig. 9 and Table S14-S22).

Overall, *TaPEX11* genes exhibited gene- and condition-specific expression patterns. Across developmental stages, *TaPEX11-7* and *TaPEX11-10* showed moderate and relatively stable expression in most tissues, whereas *TaPEX11-8*, *TaPEX11-9*, and *TaPEX11-12* were preferentially expressed in leaf, stem, and spike tissues, with increased expression at later developmental stages. The remaining genes displayed generally low expression throughout development (Fig. 9A).

Tissue-specific analysis further highlighted distinct expression preferences. *TaPEX11-7* and *TaPEX11-10* were highly expressed in root, stem, and grain, while *TaPEX11-8*, *TaPEX11-9*, and *TaPEX11-12* showed leaf-specific enrichment (Fig. 9B). During reproductive development, several genes—particularly *TaPEX11-7*, *TaPEX11-10*, and *TaPEX11-11*—were upregulated during spike development (Fig. 9C). During grain filling, *TaPEX11-7* and *TaPEX11-10* exhibited higher expression at early stages, especially in the aleurone tissue, while *TaPEX11-1* and *TaPEX11-11* showed moderate expression (Fig. 9D).

Under abiotic stress conditions, *TaPEX11* genes displayed distinct stress-responsive behaviours. Following salt stress, *TaPEX11-7*, *TaPEX11-10*, and *TaPEX11-11* were highly expressed under control conditions but showed reduced expression under NaCl treatment, whereas *TaPEX11-1* was transiently induced at early time points (Fig. 9E). Under osmotic stress (PEG6000), *TaPEX11-4* showed strong and consistent upregulation across genotypes and time points, while *TaPEX11-2* and *TaPEX11-3* displayed moderate, genotype-dependent induction (Fig. 9F). Notably, *TaPEX11-5* and *TaPEX11-6* exhibited genotype-specific responses, with marked induction in Giza 168 but minimal expression in Gemmiza 10, suggesting differential osmotic stress sensitivity. In Fig. 9G, In heat stress experiments, heat-tolerant genotypes exhibited higher expression of *TaPEX11-2* and *TaPEX11-4* compared with heat-sensitive lines, indicating a potential role in thermotolerance.

Under combined heat and drought stress, *TaPEX11-4* showed the strongest induction, particularly after prolonged exposure, while *TaPEX11-1* and *TaPEX11-3* also exhibited time-dependent upregulation (Fig. 9H). In contrast, most other *TaPEX11* genes displayed low or weak responsiveness to these stresses. Biotic stress analysis revealed that *TaPEX11-1*, *TaPEX11-3*, and *TaPEX11-11* were early induced following stripe rust infection, whereas *TaPEX11-4* and *TaPEX11-6* responded at later infection stages, indicating differential roles during pathogen response (Fig. 9I). Collectively, these results demonstrate that *TaPEX11* genes exhibit distinct tissue-specific and stress-responsive expression patterns, with a subset of genes—particularly *TaPEX11-4*, *TaPEX11-7*, and *TaPEX11-10*—likely playing prominent roles in developmental regulation and abiotic and biotic stress adaptation in wheat.

### Hydrogen peroxide production and catalase activity

**Fig. 10:**
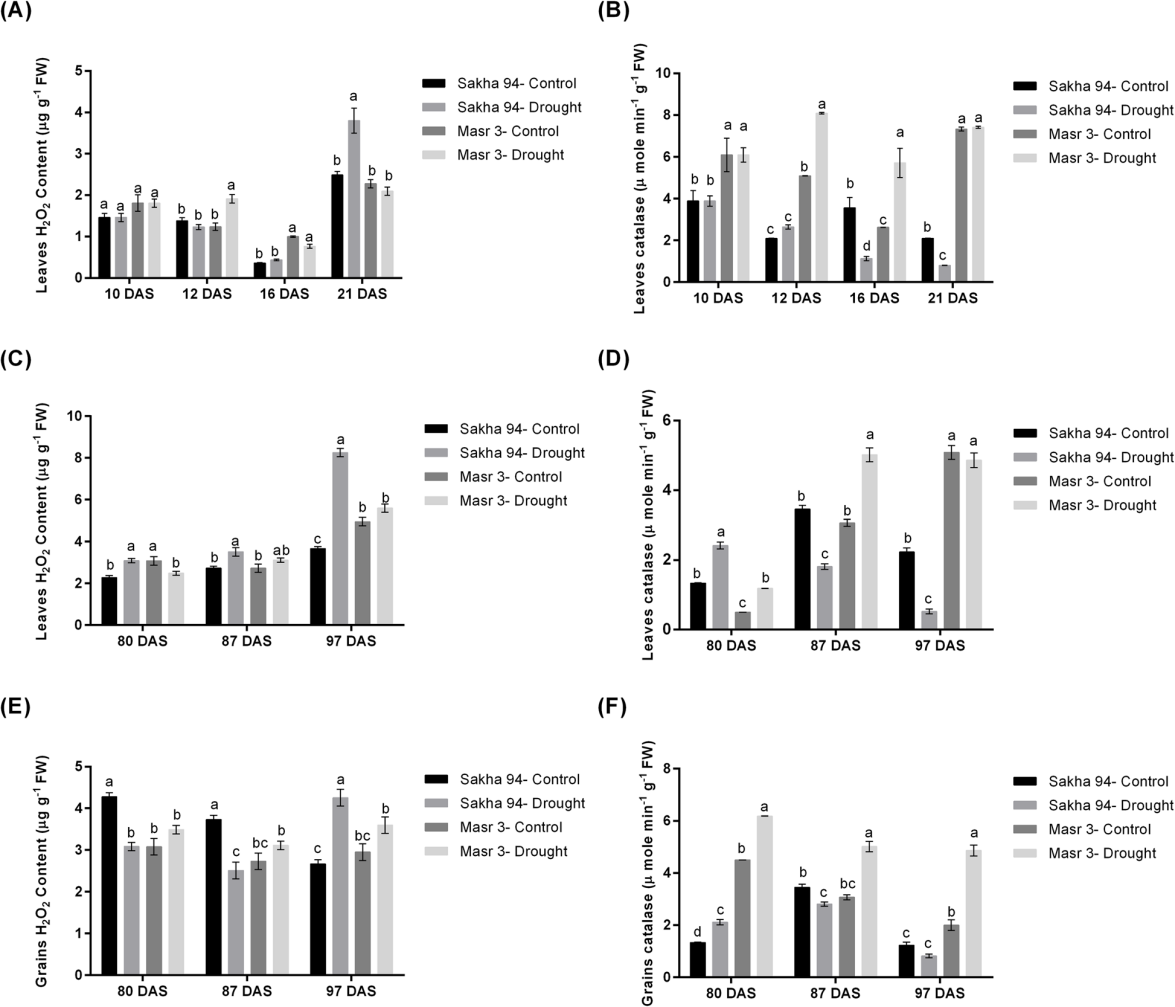
Hydrogen peroxide (H_2_O_2_) concentration and catalase enzyme activity in two wheat genotypes (Sakha 94 and Masr 3) during vegetative and reproductive stages under irrigated and drought conditions. **A** Leaves H_2_O_2_ concentration (µg g^−1^ FW) under control and drought conditions at 10, 12, 16, and 21 days after sowing (DAS). **B** Catalase activity (µ mole min^−1^ g^−1^ FW) in leaves under control and drought conditions at 10, 12, 16, and 21 DAS. **C** Leaves H_2_O_2_ concentration (µg g^−1^ FW) under control and drought conditions at 80, 87, and 97 DAS. **D** Catalase activity (µ mole min^−1^ g^−1^ FW) in leaves under control and drought conditions at 80, 87, and 97 DAS. **E** Grains H_2_O_2_ concentration (µg g^−1^ FW) under control and drought conditions at 80, 87, and 97 DAS. **F** Catalase activity (µ mole min^−1^ g.^−1^ FW) in grains under control and drought conditions at 80, 87, and 97 DAS. The data represents the mean of three biological replicates. The least significant difference (LSD) test with a *P* < 0.05 was used to compare the treatments and identify any significant effects. Different letters on bars indicate significance at *P* < 0.05 (small letters for comparison of individual)

To investigate the activities of oxidative stress in wheat, hydrogen peroxide (H₂O₂) content and peroxisomal catalase (CAT) antioxidant enzyme activities were measured in leaves and grains of two genotypes (Sakha 94 and Masr 3) grown under irrigated circumstances (control) and drought conditions during both vegetative (in growth chamber) and reproductive stages (under field conditions) (Fig. 10). During the vegetative stage, leaves of the Masr 3 genotypes had significantly higher H₂O₂ accumulation levels at 16 DAS under drought conditions than did those of Sakha 94 (Fig. 10). During the vegetative stage, H₂O₂ levels in leaves in Masr 3 exhibited significantly higher H₂O₂ accumulation than Sakha 94 at 16 DAS under drought (Fig. 10A). Contrarily, at 21 DAS, H₂O₂ elevated significantly in Sakha 94 under drought stress. Surprisingly, CAT activity (Fig. 10B) increased under drought in Masr 3 and declined in Sakha 94 at 16 and 21 DAS. In the reproductive stage under field conditions, H₂O₂ levels in leaves (Fig. 10C) remained significantly elevated under drought stress in Sakha 94. CAT activity in leaves (Fig. 10D) increased under drought in both genotypes, with Sakha 94 again showing significantly higher activity, particularly at 80 DAS but declined at 87 and 97 DAS. In contrast, Masr 3 progressively increased CAT activity throughout intervals with higher activity at 80 and 87 DAS under drought stress. In grains, drought stress also triggered a marked decline in H₂O₂ content (Fig. 10E), especially at 80 and 87 DAS in Sakha 94. At 97 DAS, drought induced elevation of H₂O₂ levels in both genotypes with Sakha 94 exhibiting significantly higher levels than Masr 3. In parallel, CAT activity in grains (Fig. 10F) was induced under drought, but Masr 3 displayed a significantly higher activity than Sakha 94 at most time points.

### Gene expression of *TaPEX11* genes by qRT-PCR

**Fig. 11:**
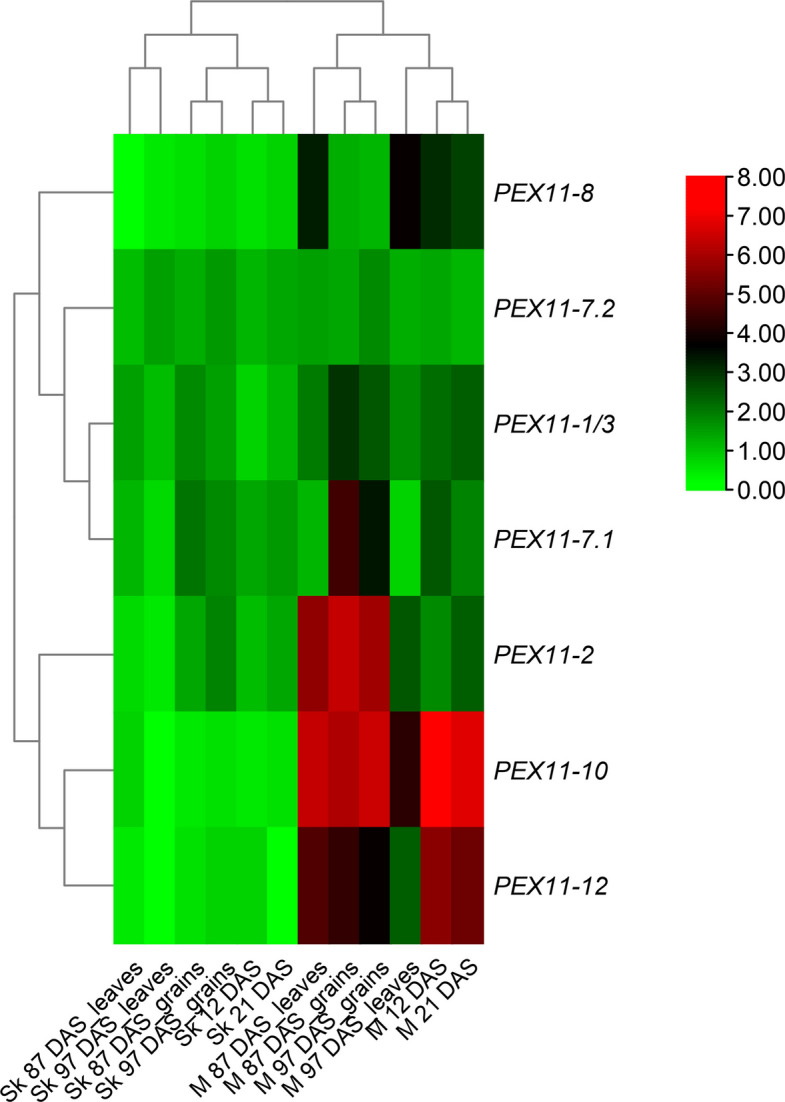
Heatmaps representing the gene expression of *TaPEX11* homologous genes as fold change (FC) in wheat genotypes (Sakha 94 and Masr 3) in response to drought stress

**Fig. 12:**
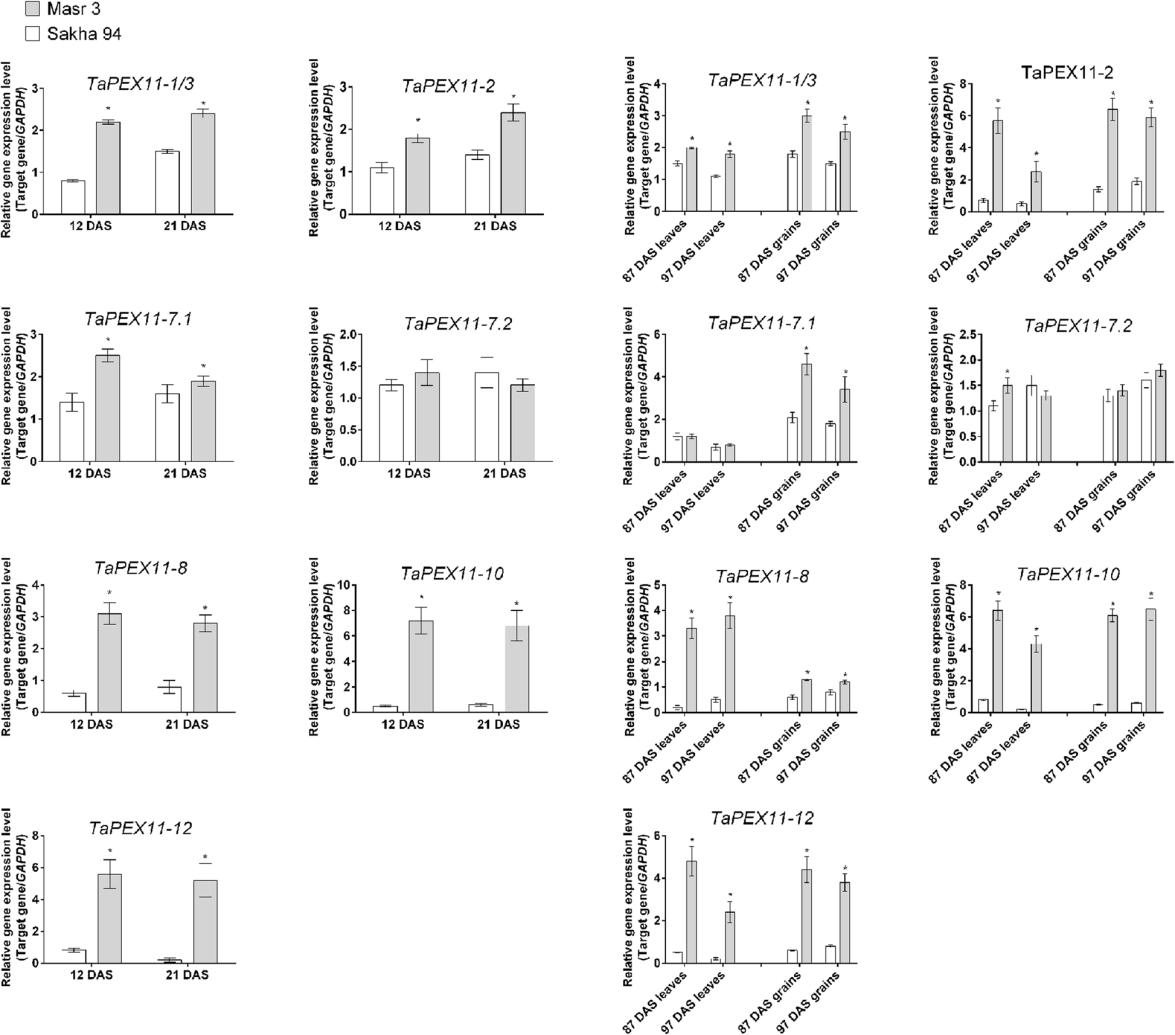
Relatively expressed *TaPEX11* genes in two wheat genotypes, Sakha 94 and Masr 3 in response to drought stress: qRT-PCR confirmation of genotype-specific drought induction patterns predicted by transcriptome analysis

To validate in silico gene expression analysis of *TaPEX11* genes in transcriptome datasets, we analyzed gene expression in two different genotypes contrasting in their tolerance to stress (Sakha 94 as less tolerant and Masr 3 as more tolerant) by quantitative real time PCR using specific primers for seven *TaPEX11* genes. We used both genotypes in two different experiments: the first under controlled growth conditions after 12 DAS and 21 DAS for evaluating *TaPEX11* gene expression in response to drought stress during vegetative stage, the second experiment was under field conditions evaluated *TaPEX11* expression in leaves and grains of both genotypes under drought stress during the reproductive stage (87 and 97 DAS). Figure 11 showing heatmap for gene expression results by qRT-PCR in both genotypes relative to control value in each sample (the results from three biological replica and three technical replica). The results showed that gene expression of *TaPEX11* genes in Masr 3 was higher than (> 2 FC) in response to drought in Sakha 97 in most samples (Fig. 12). In response to drought stress at vegetative stage, Masr 3 consistently shows higher induction of several *TaPEX11* genes under drought stress compared to Sakha 94, especially at 21 DAS. Six *TaPEX11* (*TaPEX11*−1/3, *TaPEX11-*2, *TaPEX11-*7*.1*, *TaPEX11*−8, *TaPEX11*−10, *TaPEX11*−12) genes showed significantly higher expression in Masr 3, indicating a stronger and perhaps earlier transcriptional activation in response to drought. Sakha 94 shows delayed or lower expression levels, possibly reflecting a weaker stress response mechanism. In response to drought stress at reproductive stage, *TaPEX11* gene expression remained consistently higher in leaves of Masr 3 than Sakha 94, particularly at 87 DAS, suggesting persistent activation under prolonged field drought stress. In grains, several *TaPEX11* genes showed enhanced expression in Masr 3 particularly at 87 DAS, indicating a possible role in stress mitigation during seed development. The gene expression in Sakha 94 is more variable and generally lower, which may be correlated with reduced drought resilience and grain quality under stress. *TaPEX11-8, TaPEX11-10,* and *TaPEX11-12* down-regulated in leaves particularly at 97 DAS, while *TaPEX11-1/3* up-regulated in grains at 87 DAS and *TaPEX11-7.1* up-regulated in grains at 87 and 97 DAS. *TaPEX11-7.2* did not show any significant change in both genotypes in both experiments.

Gene expression values as fold change are the mean of three different biological and three technical replica of each sample and were normalized to Red indicates high expression, black indicates moderate expression, and green indicates low expression. Values of FC ± SD are provided in Fig. 12.

The relative transcript levels were normalized against *GAPDH* and are shown as fold changes relative to the control. The least significant difference (LSD) test with a *P* < 0.05 was used to compare the treatments and identify any significant effects. Asterisks indicate significant differences between control and drought treatments within the same genotype (*P* < 0.05). Error bars display standard deviation of the mean of three biological and three technical replicas (SD).

## Discussion

Although the *PEX11* gene family has been thoroughly examined in the model plant *A. thaliana* [4], it has not received enough attention in crops, especially wheat (*Triticum aestivum* L.). This study explores structural, functional aspects of *PEX11* in wheat as well as genomic organization of *PEX11* gene family and its regulation under drought stress.

### Structural conservation and diversification of wheat *PEX11* genes suggest functional specialization

The presence of highly conserved regions across *TaPEX11* homologs likely corresponds to structural or functional motifs necessary for peroxisome biogenesis, such as transmembrane helices or sites mediating protein–protein interactions [11]. All the identified TaPEX11 proteins contained the pfam05648 domain, emphasizing further their function in membrane elongation and fission [23]. With a narrow range of isoelectric points and molecular weights among homologs, structural conservation could be important for interactions either with acidic phospholipid membranes or binding partners [11]. Predicted stability index less than 40-strongly supports their essentiality in the cell since unstable proteins are usually eliminated during purifying selection [24]. Through motif analysis, 10 conserved motifs were revealed, of which three motifs were PEX11-specific. These are likely to be involved in core functions such as membrane binding or oligomerization, as shown in *Arabidopsis* [25]. Phylogenetic analysis revealed that the wheat *PEX11* gene family has undergone significant expansion, forming four strongly supported clades indicative of lineage-specific duplication events. Such expansion is characteristic of polyploid genomes and often provides the genetic basis for subfunctionalization or neofunctionalization. Lineage-specific clustering is in agreement with the gene duplication events widely observed in polyploid species such as wheat, where genome triplication often drives gene family expansions [26]. The close association between phylogenetic clades, conserved motif architecture, and gene structure indicates that the wheat PEX11 family has experienced both functional conservation and divergence. Clades 1 and 2 exhibit conserved motifs and intronless structures, consistent with retention of core PEX11 functions, whereas clades 3 and 4 display complex exon–intron organization and distinct motif patterns, suggesting regulatory diversification and subfunctionalization following gene duplication. Together, these integrated analyses confirm that TaPEX11 clades represent evolutionarily and functionally distinct subfamilies. The conserved clustering of wheat, rice, and *Arabidopsis* PEX11 proteins within defined clades, supported by high bootstrap values, suggests that core PEX11 functions have been retained throughout angiosperm evolution. Co-clustering of rice OsPEX11 proteins along with TaPEX11 homologs into four clades evidence that there might be a conservation of functional roles in monocots retained from their common ancestor [27].

The three-dimensional structural models of wheat PEX11 proteins indicated presence of TM domains, a characteristic feature that agrees with their established role in peroxisomal membrane elongation, fission, and proliferation [23, 28]. A conserved N-terminal amphipathic helix, Pex11-Amph, is essential for peroxisome fission and membrane remodelling [29]. This helix induces tubulation upon interactions in vitro with liposomes, mainly occurring in negatively charged vesicles considered closest to peroxisomal membranes [29]. These correspond to the alpha-helical regions that form the core structural framework of these proteins, which agrees with previous observations in *Arabidopsis* and yeast PEX11 homologs where alpha-helices are critical for membrane integration and oligomerization [29–31]. Spatial variability in the arrangement of helices and loop regions among the TaPEX11 isoforms points toward structural diversification, which may underpin functional specialization within this protein family. This may reflect adaptations to different regulatory or environmental cues, as suggested for the PEX11 isoforms in other plant species [25]. Only experimentally resolved membrane-protein templates containing annotated transmembrane helices were selected to maximize biological relevance; however, structural predictions were interpreted cautiously due to the intrinsic challenges of modelling multi-pass membrane proteins. One TaPEX11 isoform (TaPEX11-7.2) miss a detectable transmembrane segment in the canonical PEX11 architecture points to that it could act as a dominant-negative regulator through heterodimerization with membrane-bound PEX11 isoforms or it may participate in cytosolic signalling or trafficking pathways associated with peroxisome biogenesis.

Single-exon genes (*TaPEX11-1* to *TaPEX11-6*) may contribute to enhanced transcriptional efficiency under stress conditions [32]. While *TaPEX11-8* to *TaPEX11-12*, were multi-exons. This may reflect a balance between conserved, housekeeping roles-assumed by single-exon isoforms-and more specialized functions that require complex regulation multi-exon isoforms. Two variants of TraesCS4A02G442900 suggesting post-transcriptional regulation via alternative splicing and regulatory plasticity in this family, which could confer rapid adaptation to metabolic or environmental cues [33–35].

### Genomic distribution and evolutionary conservation of *TaPEX11* genes reflect functional stability and adaptive potential in wheat

The chromosomal localization of *TaPEX11* genes on nine chromosomes may arise from segmental duplications, a key driver of gene family expansion in polyploids [36]. The localization of *TaPEX11-8* to *TaPEX11-12* near the chromosomal termini might be indicative of a high rate of recombination and is commonly associated with adaptive evolution as reported by Huang et al. [37]. These subtelomeric regions are also hotspots for transposable elements and dynamic rearrangements, thus contributing to structural diversification in the *PEX11* family [38]. The identification of paralogous pairs indicates segmental duplication events, a hallmark of gene family expansion in polyploids [36]. It is likely that these duplications accommodated subfunctionalization or neofunctionalization, allowing wheat to adapt wide environmental stresses without losing the fundamental core peroxisomal functions. For example, duplicated *PEX11* genes in *Arabidopsis* partitioned for peroxisome proliferations and various stress responses [25], a pattern that may extend in wheat. The present study revealed that the *TaPEX11* gene family in wheat consists of 12 loci encoding 13 proteins, a number lower than the theoretical expectation (~ 15) for a hexaploid genome. This discrepancy can be attributed to gene loss or pseudogenization during subgenome evolution but also to the stringent criteria applied for gene identification.

The ratio of Ka and Ks can determine whether there is selective pressure on the coding gene of this protein [39]. Purifying selection may be acting to remove unstable, non-functional variants from the genome as unstable proteins. Segmental duplications are common in polyploid species like wheat, however, they operate in concert with whole-genome duplication (WGD) events and subsequent diploidization that collectively drive gene family diversification [40, 41]. For instance, duplications between chromosomes 4A-4B-5A, 2A-2D, and 7A-7D probably a combination of ancestral WGDs and homoeologous chromosome evolution during wheat evolution according to the "two-step" hexaploidization model [26], rather than segmental duplication alone. The comparative syntenic analysis showed strong conservation of *PEX11* orthologs in wheat and rice, sorghum, and maize. The 12–14 orthologous pairs identified in each comparison suggest that *PEX11* genes have been conserved since the divergence of monocots ~ 100 million years ago [42]. Such synteny is typical for genes taking part in the fundamental processes of cellular metabolism [43].

### Regulatory elements and functional enrichment of *TaPEX11* genes reveal coordinated stress responses and developmental specificity in wheat

Studying *cis*-regulatory elements (CREs) in the *PEX11* promoter regions has enlightened their relative regulatory mechanism and functional evolution. The diverse elements with tissue-specificity, hormone responsiveness, and stress adaptation suggest the precisions with which they operate *TaPEX11* genes in development and environmental responses, consistent with their involvement in peroxisome proliferation and stress resilience [27].

The endosperm-specific element in *TaPEX11-4* and *TaPEX11-5* may relate to seed development or processes relating to lipid metabolism, which are critical during germination [44]. *TaPEX11-4* possesses no developmental elements aside from endosperm motifs, which suggests that the gene has a special role in seed-linked processes. Circadian-specific elements in *TaPEX11-7* and *TaPEX11-8* are further indications that expression is regulated throughout the diurnal cycle, synchronizing the peroxisomal functioning with metabolic cycles, as evident from stress responses that have been found as regulated by circadian rhythms [45]. Abundance of the hormone responsive CREs consisted of ABRE, AuxRE, and GARE to emphasize *TaPEX11* genes' involvement within the signalling network of phytohormones. It is the only one except *TaPEX11-4* that has in its promoter SARE, which indicates the relevance in SA-related defences like pathogen resistance [46]. The heterogeneity within the hormone-related motifs in different promoters would reflect functional divergence, allowing hormonal crosstalk under different stress conditions or developmental cues [47]. The prevalence of these stress-related CREs, including DRE, STRE, and WUN, proves that *TaPEX11* genes are functional in the abiotic stress tolerance phenomenon. A great amount of STRE in *TaPEX11* can be seen as having linked functionality to these genes during stress responses in organisms with peroxisome functions in oxidative stress and MBS and ARE motifs further support their association in cross-tolerance mechanisms, where drought and oxidative stress converge on the same pathways [48]. The dominance of as1 (root-specific) elements within *PEX11-1*, *−11*, and *−12* argues robust expression in root tissues and probably further aids soil stresses or possibly root-specific peroxisome function [49]. The differences in the hormone motif densities especially ABRE compared to TGACG suggest a possible sensitivity of paralogs to different hormones and thus fine-tuning of the response to development and environmental stimuli. The high STRE frequency in certain genes indicates subfunctionalization of the *TaPEX11* family for specialized adaptation roles to stress.

Gene Ontology (GO) enrichment analysis is widely used to interpret large-scale genomic data [50, 51]. In this study, GO enrichment of *TaPEX11*-associated genes highlighted key processes related to peroxisome regulation, redox homeostasis, and stress responses, underscoring the functional importance of *TaPEX11* genes in maintaining cellular homeostasis. The enrichment of terms such as regulation of peroxisome size, response to high light intensity, and oxidoreductase-related activities supports the established role of PEX11 proteins in peroxisome proliferation and fission, processes that are critical for adapting peroxisomal metabolism to environmental stress [52]. Notably, enrichment of photosynthesis- and chloroplast-associated GO terms likely reflects indirect but biologically meaningful links between peroxisomes and chloroplasts. Peroxisomes are integral to photorespiration and ROS detoxification, processes tightly coupled to photosynthetic activity, especially under drought and high-light stress [12]. Thus, PEX11-mediated modulation of peroxisome abundance and functionality may influence chloroplast performance and energy balance by regulating ROS signalling and metabolite fluxes. Together, these findings suggest that *TaPEX11* genes act as key regulators of peroxisome dynamics that integrate stress signalling, redox control, and energy metabolism, thereby enhancing wheat adaptability to adverse environmental conditions.

Integration of TF enrichment analysis also identified putative upstream regulators of *TaPEX11* genes, highlighting members of the *AP2-ERF* family as important nodes in the network. Ap2/ERF transcription factor families are central to numerous plant responses to abiotic stresses and hormone signalling [53–56]. AP2/ERFs interact with other transcription-factor families, such as WRKYs, bHLHs, and MYBs, in enhancing cold stress tolerance [54]. Combined, these results give weight to a model in which *TaPEX11* genes are incorporated into a gene regulatory network coordinated by key stress-associated TFs, especially *AP2-ERF* and *bHLH*, to bring about stress tolerance mechanisms in wheat. Future directions should aim to clarify the precise mechanisms of these TFs and their interaction within the gene network at large, paving the way to understand plant adaptability and resilience in changing environments.

### Genotype-specific activation and temporal expression of *TaPEX11* genes highlight peroxisomal redox dynamics in drought tolerance

Effective drought tolerance depends on the quick sensing of oxidative imbalance by the plant and mobilising the appropriate antioxidant response [57–59]. The converse variations of H₂O₂ accumulation and peroxisomal CAT activity exhibited by Sakha 94 and Masr 3 seem to typify some major temporal and tissue-specific differences that account for their differential behaviour under stress. During early vegetative growth, Masr 3 had an H₂O₂ surge accompanied by a high increase in CAT activity. This early oxidative 'signal' could be a priming event that triggers the redox-sensitive transcription factors and antioxidant machinery prior to extensive cellular damage [57–59]. On the other hand, Sakha 94 had a delayed ROS peak with a depressed CAT activity, representing slower stress perception and impaired detoxification ability. Peroxisomes act as the central hubs for peaking both the production and detoxification of ROS, so a rapid peroxisome multiplication with upregulated catalase would offer double protection in preventing excessive oxidative damages while still preserving the signalling functions of well-regulated H₂O₂ bursts.

Transcriptome data analysis revealed three functionally expressed clusters: (i) constitutive "maintenance" isoforms (*TaPEX11-7, −10, −11*) to maintain basal peroxisome homeostasis; (ii) rapid-stress sentinels (*TaPEX11-1, −3, −5, −6*) that respond within hours to salt, osmotic, heat, and biotic triggers; (iii) late or sustained responders (*TaPEX11-4, −8, −9, −11, −12*) that modify peroxisome abundance during grain fill and extended abiotic stress. These expression signatures, in conjunction with distribution of *Cis* elements and GO enrichment for photosynthesis, peroxisome morphogenesis and redox metabolism, suggest an overarching role for the family in balancing energy production with reactive oxygen signalling during growth and defence.

The qPCR experiment comparing the drought-tolerant cultivar Masr 3 with the sensitive Sakha 94 validates the *in-silico* predictions at the genotype level through the data. Masr 3, the tolerant one, was very consistent in the fact that it directly activated earlier and stronger six stress-responsive homeologs (*TaPEX11-1, −2, −3, −7.1, −8, −10, −12*) at the vegetative stage and it also kept the increased transcript amount in leaves and grains during the reproductive drought phase. Peroxisomes play a crucial role in plant stress responses, with peroxisomal proteins showing differential expression under various abiotic stresses [1, 60]. The genotype dependence revealed in the qPCR experiment supports the differential cultivar-specific patterns, which were identified from the transcriptome datasets for osmotic and heat stress and strengthens the notion that the efficient activation of key *TaPEX11* isoforms is one of the main characteristics of stress-tolerant germplasm. The constitutive pair *TaPEX11-7/10* that is down-regulated under drought in flag leaves was however up-regulated in grains of Masr 3, which signals tissue-specific prioritisation of peroxisome activity during seed filling. *TaPEX11-7.2* hardly changed in both experiments, which implies it might be carrying out a housekeeping or redundant function, whereas *TaPEX11-12* displayed a very strong but transient activation pattern that could be associated with the oxidative burst that comes with early grain development. Such differential kinetics provide evidence for the sub-functionalisation model which has been deduced from the transcriptomic atlas.

Although the current validation focused on drought stress, transcriptome evidence suggests that *TaPEX11* genes also respond to salt, heat, and pathogen stress. Future studies will include experimental validation under multiple abiotic and biotic stresses to clarify these broader functions.

## Conclusion

The study conducted the first complete genome-wide characterization of the *PEX11* gene family in hexaploid wheat revealing 12 *TaPEX11* genes shaped by segmental duplications and strong purifying selection and demonstrating their structural and functional diversification through stress-responsive promoter elements, tissue-specific expression, and genotype-dependent induction under drought. Crucially, drought-tolerant wheat (Masr 3) showed coordinated upregulation of key *TaPEX11* isoforms, elevated catalase activity, and enhanced oxidative stress management revealing a mechanistic link between peroxisome dynamics and abiotic stress resilience. Evolutionary analyses uncovered conserved synteny with rice, sorghum, and maize, highlighting preserved functions across cereals. The present study lays a foundation for future functional validation through transgenic or CRISPR/Cas-based approaches to confirm the roles of *TaPEX11* genes in drought tolerance (Lines 905–910). The integrated approach combining genomic, transcriptomic, and biochemical validation establishes *TaPEX11* genes as regulatory nodes in stress adaptation while providing a curated genetic resource for the plant research community. These findings advance understanding of peroxisome-mediated stress adaptation in polyploid crops and offer targets for improving environmental resilience in cereals.

## Supplementary Information

Supplementary Material 1.

Supplementary Material 2.

## Data Availability

The data and datasets used and/or analyzed in the current study are available from the following repositories: the International Wheat Genome Sequencing Consortium (IWGSC) genome assembly at EnsemblPlants (https:/plants.ensembl.org/Triticum_aestivum/Info/Index), WheatOmics 1.0 (http:/wheatomics.sdau.edu.cn/coexpression/index.html), Wheat URGI (Unité de Recherche Génomique-Info) (http:/www.wheat-expression.com/), and the NCBI Gene Expression Omnibus (https:/www.ncbi.nlm.nih.gov/geo/). Accession numbers for the transcriptome datasets used in this study are provided in Table S5, and the corresponding analyses are listed in Tables S12–S20. Specifically, datasets from project PRJEB25639 (WheatOmics 1.0) were used for co-expression analysis. All data supporting the findings of this study are available in the main text and supplementary information.

## Abbreviation

PEX: Peroxin
*TaPEX11*: *Triticum aestivum* Peroxin 11
SRA: Sequence Read Archive
TPM: Transcript Per Million
FC: Fold Change
LSD: Least significant difference
CREs: *Cis*-Acting regulatory elements
