# Supplementary material for: Integrative gene duplication and genome-wide analysis characterize Peroxin11 gene family in wheat
Source: BMC Genomics. 2026 Apr 11;27:369. doi: 10.1186/s12864-026-12771-2 (PMC13072609; doi:10.1186/s12864-026-12771-2)
Supplement: Supplementary file 1 — Supplementary Material 1. [file 12864_2026_12771_MOESM1_ESM.zip › Table S4.docx]

**Table S4**: Template selection and quality assessment for homology-based 3D modelling of TaPEX11 proteins.

| **TaPEX11 proteins** | **Template ID** | **Oligo-state** | **GMQE** | **Seq Identity** | **Coverage** |
| --- | --- | --- | --- | --- | --- |
| TaPEX11-1 | A2XFQ8.1.A | monomer | 0.90 | 85.84 | 0.98 |
| TaPEX11-2 | A2XFQ8.1.A | monomer | 0.90 | 84.55 | 0.98 |
| TaPEX11-3 | A2XFQ8.1.A | monomer | 0.90 | 85.84 | 0.98 |
| TaPEX11-4 | A0A3B6B243.1.A | monomer | 0.92 | 94.76 | 1.00 |
| TaPEX11-5 | A0A3B6B243.1.A | monomer | 0.92 | 95.61 | 1.00 |
| TaPEX11-6 | A0A3B6B243.1.A | monomer | 0.92 | 100.00 | 1.00 |
| TaPEX11-7.1 | Q5VRJ8.1.A | monomer | 0.94 | 90.13 | 1.00 |
| TaPEX11-7.2 | Q5VRJ8.1.A | monomer | 0.92 | 90.29 | 1.00 |
| TaPEX11-8 | Q10SM7.1.A | monomer | 0.93 | 87.34 | 1.00 |
| TaPEX11-9 | Q10SM7.1.A | monomer | 0.93 | 88.19 | 1.00 |
| TaPEX11-10 | Q5VRJ8.1.A | monomer | 0.94 | 89.27 | 1.00 |
| TaPEX11-11 | Q5VRJ8.1.A | monomer | 0.94 | 89.70 | 1.00 |
| TaPEX11-12 | Q10SM7.1.A | monomer | 0.93 | 86.08 | 1.00 |
