# Supplementary material for: Integrative gene duplication and genome-wide analysis characterize Peroxin11 gene family in wheat
Source: BMC Genomics. 2026 Apr 11;27:369. doi: 10.1186/s12864-026-12771-2 (PMC13072609; doi:10.1186/s12864-026-12771-2)
Supplement: Supplementary file 1 — Supplementary Material 1. [file 12864_2026_12771_MOESM1_ESM.zip › Table S7.docx]

**Table S7**: Soil water content (%) under control and drought conditions at different sampling time points for the wheat cultivars Sakha 94 and Masr 3. Values represent mean ± standard error (SE) from replicated plots.

| **Sampling time point** | **Sakha 94** | | **Masr 3** | |
| --- | --- | --- | --- | --- |
|  | **Control** | **Drought** | **Control** | **Drought** |
| 80 DAS | 28.1 ± 1.0 | 17.0 ± 0.8 | 27.8 ± 0.9 | 16.2 ± 0.7 |
| 87 DAS | 27.3 ± 0.9 | 14.0 ± 0.7 | 26.9 ± 0.8 | 13.1 ± 0.6 |
| 97 DAS | 26.0 ± 0.8 | 10.2 ± 0.6 | 25.6 ± 0.7 | 9.4 ± 0.5 |
