# Supplementary material for: Integrative gene duplication and genome-wide analysis characterize Peroxin11 gene family in wheat
Source: BMC Genomics. 2026 Apr 11;27:369. doi: 10.1186/s12864-026-12771-2 (PMC13072609; doi:10.1186/s12864-026-12771-2)
Supplement: Supplementary file 1 — Supplementary Material 1. [file 12864_2026_12771_MOESM1_ESM.zip › Table S8.docx]

**Table S1: Primer sequences used in the study for wheat *PEX11* genes.**

| **Name** | **Primer pairs** | **Sequence (5'->3')** | **Length** | **Tm** | **GC%** | **Product size** |
| --- | --- | --- | --- | --- | --- | --- |
| ***TaPEX11-1/3***  TraesCS4D02G187900  TraesCS4B02G186600 | Fw | 5'- ACCATCAAGCTGGAAGAGGTG -3' | 21 | 60 | 52.38 | 82 |
|  | Rv | 5'- CCTCGTTCTCCTCCCCACAA-3' | 20 | 61.19 | 60 |  |
| ***TaPEX11-2***  TraesCS4A02G117800 | Fw | 5'- GTTCGTCTGGCTCGCGAAAG-3' | 20 | 61.95 | 60 | 74 |
|  | Rv | 5'- CCCAGGCGCTGAGACAC-3' | 17 | 60.09 | 70.59 |  |
| ***TaPEX11-7.1***  TraesCS4A02G442900 | Fw | 5'- AGCCTCGACATAGTTGTTGCG-3' | 21 | 61 | 52.38 | 72 |
|  | Rv | 5'- CCCTGTGACGCGAGGA-3' | 16 | 58.14 | 68.75 |  |
| ***TaPEX11-7.2***  TraesCS4A02G442900 | Fw | 5'- GCGATGCTGTCAACTTTCCT-3' | 20 | 58.84 | 50 | 80 |
|  | Rv | 5'- TGAAGCTCCGCTAGCTTGTAT-3' | 21 | 59.24 | 47.62 |  |
| ***TaPEX11-8***  TraesCS4B02G355100 | Fw | 5'- ATCAGCCCAGCCCCCAAAG-3' | 19 | 62.31 | 63.16 | 54 |
|  | Rv | 5'- AGACTTCCCAAGCAAGACGAGTG-3' | 23 | 62.67 | 52.17 |  |
| ***TaPEX11-10***  TraesCS7A02G056300 | Fw | 5'- CTGAAGAAGTCCAATGAGAGGCT-3' | 23 | 542 | 60.06 | 50 |
|  | Rv | 5'- TCAAGGCTCGATTTGATGAGAGCAA-3' | 25 | 545 | 62.82 |  |
| ***TaPEX11-12***  TraesCS5A02G524400 | Fw | 5'- TCCCTCTACTGCTGGATGACT-3' | 21 | 59.71 | 52.38 | 50 |
|  | Rv | 5'- AGCTCGACTAGACTTGCAAAGA-3' | 22 | 59.44 | 45.45 |  |
| Ref gene  **GAPDH** | Fw | 5'-TTGCTCTGAACGACCATTTC-3' | 20 | 60.7 | 45 | 175 |
|  | Rv | 5'-GACACCATCCACATTTATTCTTC-3' | 23 | 59.8 | 39.1 |  |
