# Supplementary figures and images for: Integrative gene duplication and genome-wide analysis characterize Peroxin11 gene family in wheat
Source: BMC Genomics. 2026 Apr 11;27:369. doi: 10.1186/s12864-026-12771-2 (PMC13072609; doi:10.1186/s12864-026-12771-2)

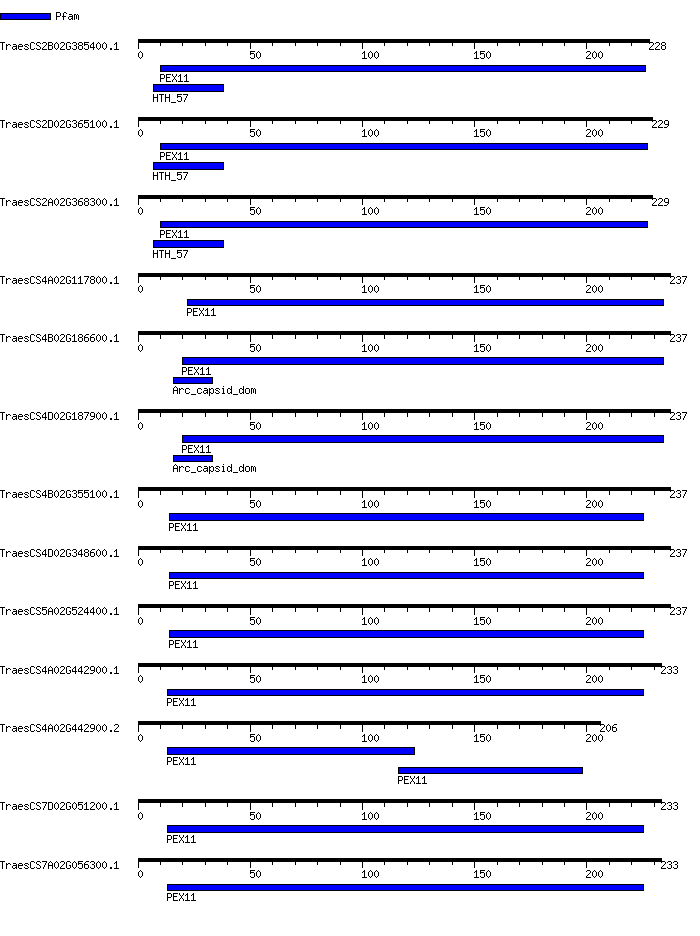


Figure S1: Identification of PEX11 domain in CD-NCBI.

Supplement: Supplementary file 2 — Supplementary Material 2. [file 12864_2026_12771_MOESM2_ESM.zip › Figure S1.docx]

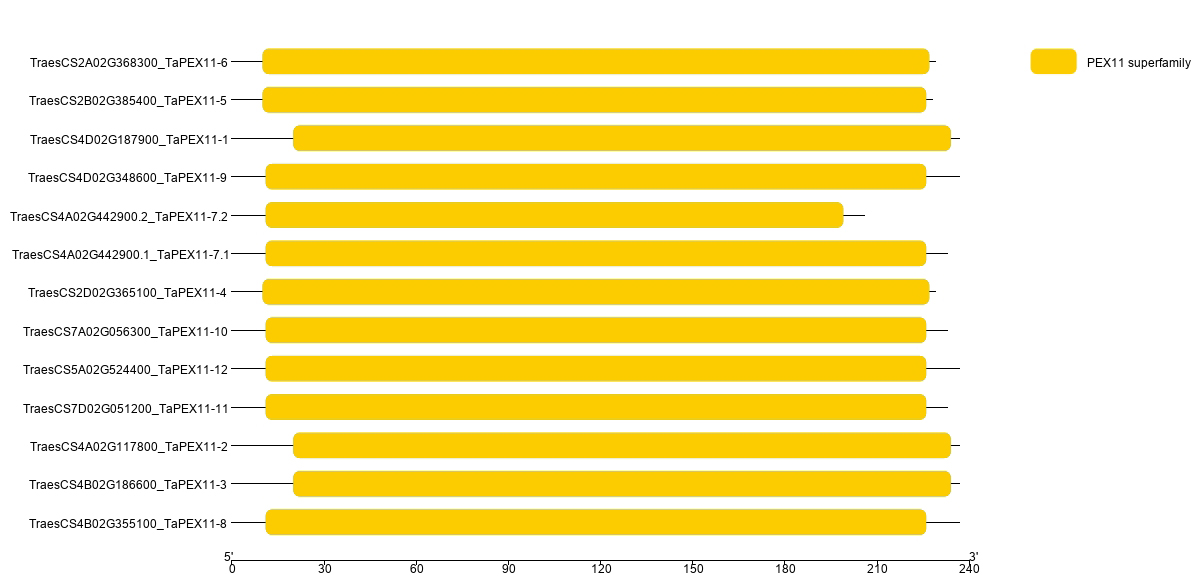


Figure S2: Identification of pfam05648 specific for peroxisomal biogenesis factor 11 Pfam.

Supplement: Supplementary file 2 — Supplementary Material 2. [file 12864_2026_12771_MOESM2_ESM.zip › Figure S2.docx]

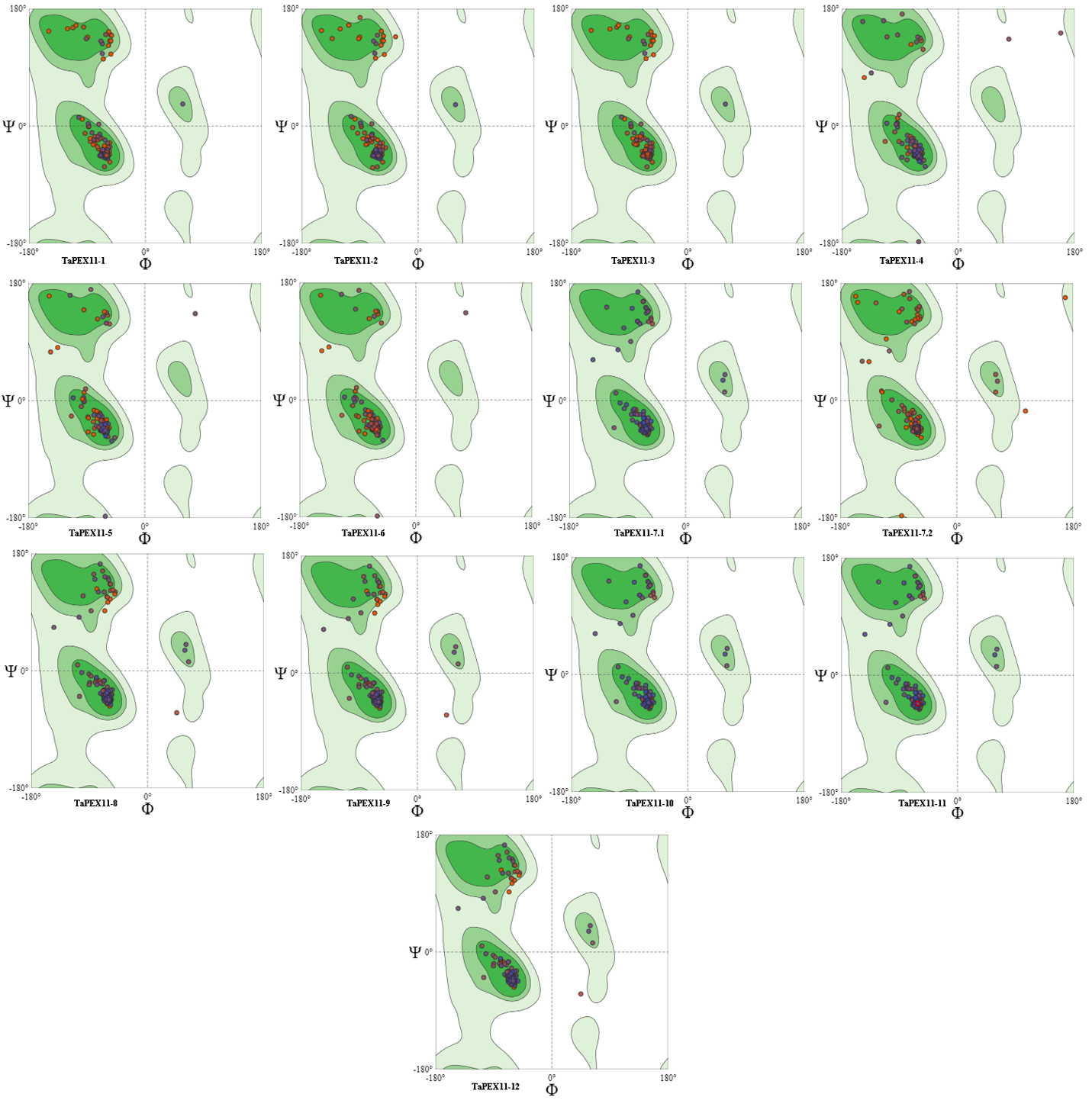


Figure S3: The Ramachandran plot for quality of TaPEX11 3D models.

Supplement: Supplementary file 2 — Supplementary Material 2. [file 12864_2026_12771_MOESM2_ESM.zip › Figure S3.docx]

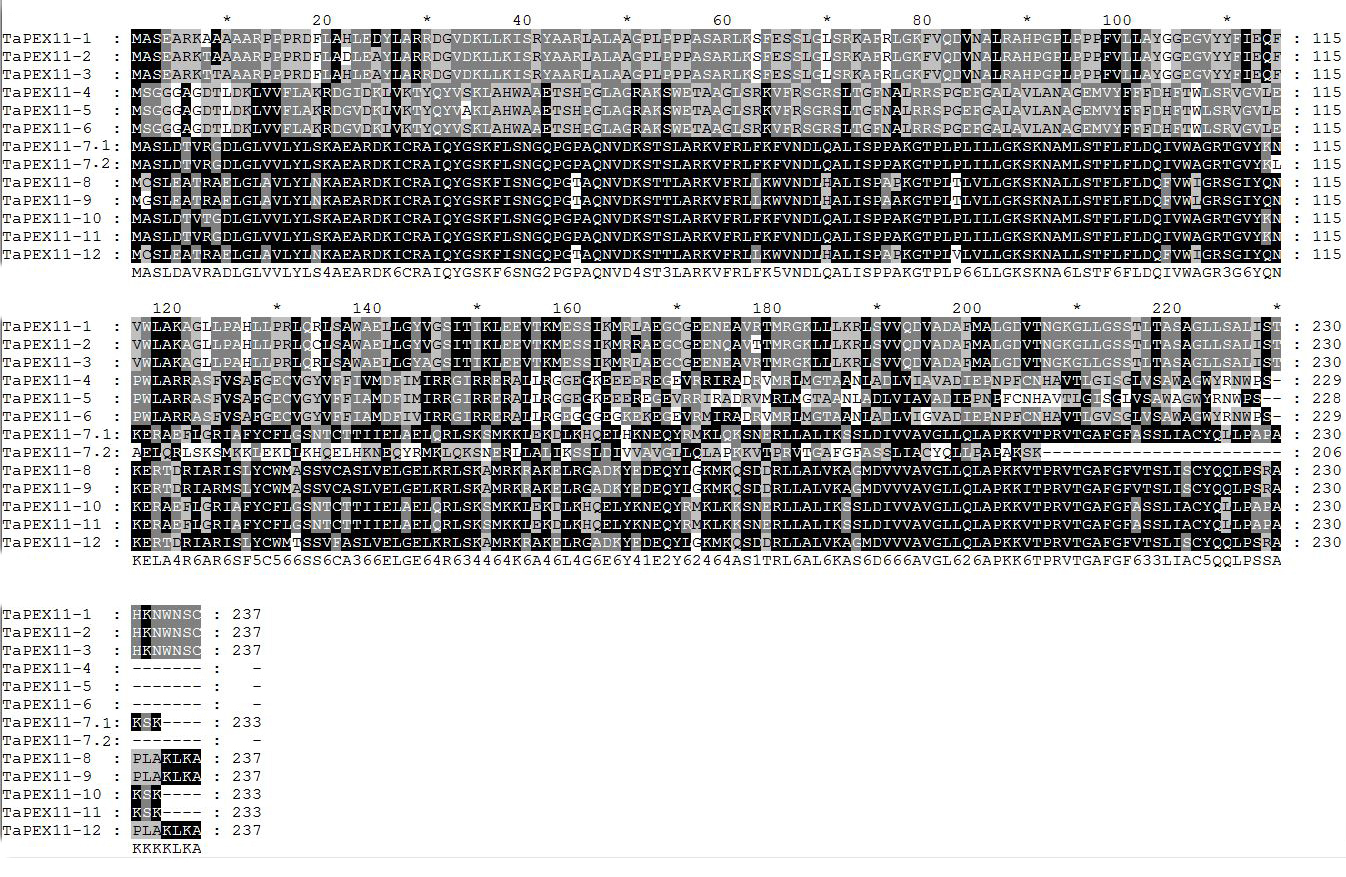


Figure S4: Multiple sequence alignment of TaPEX11 proteins.

Supplement: Supplementary file 2 — Supplementary Material 2. [file 12864_2026_12771_MOESM2_ESM.zip › Figure S4.docx]
